# Mental health indicators for children and adolescents in OECD countries: a scoping review

**DOI:** 10.3389/fpubh.2023.1303133

**Published:** 2024-02-13

**Authors:** Andreas Deckert, Silvia Runge-Ranzinger, Tobias Banaschewski, Olaf Horstick, Abdelrahman Elwishahy, Margarita Olarte-Peña, Claudia Faber, Thomas Müller, Lucia Brugnara, Julia Thom, Elvira Mauz, Diana Peitz

**Affiliations:** ^1^Heidelberg Institute of Global Health (HIGH), Heidelberg University Hospital, Heidelberg, Germany; ^2^Klinik für Psychiatrie und Psychotherapie des Kindes- und Jugendalters Zentralinstituts für Seelische Gesundheit, Mannheim, Germany; ^3^evaplan GmbH am Universitätsklinikum Heidelberg, Heidelberg, Germany; ^4^Translational Research Center, University Hospital of Psychiatry and Psychotherapy, University of Bern, Bern, Switzerland; ^5^Department of Epidemiology and Health Monitoring, Robert Koch Institute, Berlin, Germany

**Keywords:** mental health, surveillance, indicator, children, adolescence, OECD

## Abstract

**Background:**

This scoping review is a further step to build up the Mental Health Surveillance System for Germany. It summarizes and analyzes indicators used or described in Organization for Economic Co-operation and Development (OECD) countries for public mental health monitoring in children and adolescents aged 0–18 years.

**Methods:**

We searched PubMed-MEDLINE, PsycINFO, Cochrane Databases, and Google Scholar from 2000 to September 2022. The search used five general keyword categories: 1) “indicators/monitoring/surveillance” at the population level, 2) “mental/psychological,” 3) “health/disorders,” 4) “children and adolescents,” and 5) 38 OECD countries. The search was complemented with an extensive grey literature search, including OECD public health institutions and an internet search using Google. A predefined set of inclusion and exclusion criteria was applied.

**Results:**

Over 15,500 articles and documents were screened (scientific search *N =* 10,539, grey literature search more than 5,000). More than 700 articles and documents have been full-text assessed, with 382 being ultimately included. Out of 7,477 indicators extracted, an initial set of 6,426 indicators met our inclusion criteria for indicators. After consolidating duplicates and similar content, this initial set was categorized into 19 topics, resulting in a final set of 210 different indicators. The analysis highlighted an increasing interest in the topic since 2008, but indicators for the younger age, particularly those aged 0 to 2 years, were less readily available.

**Conclusion:**

Our research provides a comprehensive understanding of the current state of mental health indicators for children and adolescents, identifying both (1) indicators of public mental health noted in a previous scoping review on adults and (2) new indicators specific to this age group. These findings contribute to the development of effective public health surveillance strategies for children and adolescents and inform future research in this field.

## 1 Introduction

In accordance with the World Health Organization (WHO), *mental health* encompasses a state of mental wellbeing that empowers individuals to effectively cope with life’s pressures, realize their capabilities, excel in learning and work, and contribute positively to their communities. It serves as an essential component of overall health and wellbeing, forming the foundation for our personal and collective capacity to make decisions, nurture relationships, and shape the world in which we live. Mental health also includes the realm of positive mental health and thus goes beyond the mere absence of mental disorders. It exists along multifaceted continua, manifesting uniquely in each individual with varying degrees of challenge, distress, and potential social and clinical outcomes (1, 2). Furthermore, mental health conditions encompass a broad spectrum, including mental disorders, psychosocial disabilities, and other mental states associated with significant distress, impaired functioning, or a risk of self-harm (1).

Childhood and adolescence are developmental periods particularly susceptible to disruptive factors. More than half of mental health problems originate in childhood and adolescence and often continue into adulthood (3). Currently, almost 18% of the German population under 18 years of age live with a mental disorder (4), and more than 20% of those receive no treatment (5). Furthermore, the burden on individuals’ lives (impairment in different life domains) as well as on society as a whole (direct and indirect costs) is high (5, 6). Considering these detrimental effects, monitoring and promoting children and adolescents’ mental health possesses public health importance.

However, systematic monitoring as part of dedicated Mental Health Surveillance (MHS) strategies for children and adolescents is scarce worldwide (7). MHS is intended to systematically collect, process, and integrate data on the population’s mental health from different sources. It also involves the analysis and interpretation of these data to report results on a regular base (8). The aim of MHS is to monitor the current state and trends in public mental health and inform the initiation and evaluation of measures related to mental health prevention, promotion, care, and rehabilitation. In other words, the output of MHS should serve as a reliable empirical foundation for evidence-based policy advice, enabling political stakeholders to plan, initiate, and assess necessary health political actions (8).

The effectiveness of a health surveillance system depends on the careful selection of appropriate indicators that effectively capture the population’s mental health. Objective 4 of the WHO Comprehensive Mental Health Action Plan 2013–2030 (2021) emphasizes the need to enhance information systems, evidence, and research in the field of mental health. The plan highlights several critical pieces of information and indicators essential for a robust mental health system. This covers broad issues related to improved data collection for prevalence, risk, and protective factors for mental health and mental wellbeing, data for treatment and outcomes, data for social determinants of health in relation to mental health and mental wellbeing, and aspects of the policy framework (9).

International MHS systems often refer only to adulthood (10, 11), incorporating only isolated indicators for children and adolescents (12) or extending the existing indicators to cover adolescents from the age of 12 years and onward, as seen in the Positive Mental Health Surveillance in Canada (13). In the United States, MHS for children and adolescents is carried out by systematically assessing key metrics in summary form from different national surveys, each addressing different health issues and varying populations (14, 15).

In most countries, only a limited number of individual mental health indicators are integrated into the monitoring of overall health in children and adolescents. An example of such integration exists in Germany with the German “Health Interview and Examination Survey for Children and Adolescents” (KiGGS) study (16, 17). A more comprehensive view on mental health was made possible by the module study “BEfragung zum seeLischen WohLbefinden und VerhAlten” (BELLA study) (18) with longitudinal, nationally representative data on the mental health of this age group.

Similar to adults, the data situation in Germany on the public mental health of children and adolescents is fragmented, often cross-sectional or not representative of the population (19). Routine data from the healthcare sector are not integrated, and results from different primary data collections are not compared. There is still no regular monitoring system for the mental health of children and adolescents in Germany, such as a continuous Mental Health Surveillance System (MHS). This is why the MHS for German adults, which has been under development at the Robert Koch Institute (RKI) since 2019, should be extended by indicators specific to children and adolescents (5).

Following the process used to establish a MHS for adults (19, 20), this scoping review aims to (1) give a comprehensive overview of existing concepts and indicators and (2) synthesize existing indicators currently used in the field of public mental health of children and adolescents in the Organization for Economic Co-operation and Development (OECD) countries. This will serve as a solid foundation for developing a future core indicator set for MHS in these age groups. Specifically, the review investigates the following: 1) Which indicators related to the mental health of children and adolescents, suitable for use in public health surveillance, can be identified based on the current state of knowledge in Germany and other OECD countries? 2) What are the current scientific gaps in reporting on the mental health of children and adolescents, as reflected by unaddressed domains?

## 2 Methods

This scoping review followed the Preferred Reporting Items for Systematic Reviews and Meta-Analyzes (PRISMA) 2020 statement and its extension for scoping reviews (21). A study protocol (Supplementary material S1) was developed and aligned with previously published work on mental health indicators for adults (20). The format of a scoping review was used to present a comprehensive overview of the existing evidence in the field, to summarize the identified key concepts, and to identify gaps, irrespective of the quality of included studies (21).

### 2.1 Search strategy, databases, search terms, and inclusion criteria

The search strategy (Supplementary material S2) comprised the following three components:

1) A database-based search of academic literature (01.01.2000 to 30.09.2022) including (a) PubMed-MEDLINE,^1^ (b) Google Scholar,^2^ (c) PsycINFO,^3^ and (d) Cochrane database.^4^ The scientific searches were conducted in English, however, not excluding any language retrieved.
2) An extensive international grey literature search (01.01.2000 to 31.10.2022), including (a) Google Scholar and (b) Google search to identify and search websites of respective institutions, as well as (c) contacting public health institutions via e-mail according to the member list of the “International Association of National Public Health Institutes” (IANPHI; www.ianphi.org). The international grey literature searches were conducted in English.
3) An in-depth search was performed in German for (a) German institutions and (b) German grey literature via Google. The searches for German institutions and grey literature (in contrast to international institutions) were conducted in German.

The search strings were derived from search terms and variations/synonyms covering the following main categories: (1) “Indicators/monitoring/surveillance” at the population level, (2) “mental/psychological,” (3) “health/disorders,” (4) “children and adolescents,” and (5) “38^5^ OECD countries,” for each country individually.

For PubMed-MEDLINE, variations of terms of the same category were combined with the Boolean operator OR, then combined with the Boolean operator AND. Medical Subject Heading (MeSH) terms, free-text terms, or limitations to title and abstract were used as perceived appropriate. Due to the use of MeSH terms (most recent articles might not have been indexed yet), the search was sub-divided into two blocks, one searching for studies before 2022 using only MeSH terms and the second for studies published in 2022 using a combination of MeSH terms and free text terms (for details see Supplementary material S2). For PsycINFO for each of the PubMed search terms, the corresponding subject heading in PsycINFO was used, extended with relevant sub-categories. The “Boolean Phrase” was selected as a search strategy, and the publication date was limited. Age groups were limited manually. For the Cochrane Database, the PubMed search terms have been transferred to Cochrane and screened by title, abstract, and keywords. The publication date was limited manually. For Google Scholar, we transferred the structure and search terms from the PubMed search into 13 distinct search blocks due to the limited capacity of the search entry box, separated by countries. The records retrieved were sorted by relevance, and each block was screened by title and abstract until saturation was reached. This was in the different blocks between 80 and 120 records, and the search continued to an additional 160 records to be sure no relevant documents were missed.

For the international grey literature search, a Google search for inter- and supranational documents has been performed. In addition, all 38 OECD public health institutions of the IANPHI were screened online and additionally contacted via email, including two reminders. Two additional institutions per country (also lay organizations including non-professional or self-help groups) were added, which the authors perceived to have potential relevance or have been recommended in a snowball sampling or by the contacted institutions. The national grey literature search identified institutions/websites and documents through Google searches, which were then screened online. Preliminary included documents were then added into a separate Excel file for final full-text assessment and data extraction. Additionally, the bibliographies of the included documents were screened for relevant references.

All types of published information were screened by title and abstract (if available). Inclusion criteria were as follows: (1) focus on the mental health of the population, (2) children and/or adolescents, (3) focus on public mental health monitoring, (4) current data (date of publication after 01.01.2000), and (5) OECD countries or supranational data. Information/documents were excluded if (1) not concerned with the general population within this age group, (2) the document had no public mental health focus, (3) literature primarily centered on somatic public health (somatic conditions/non-communicable disease such as diabetes, etc.; in contrast to public mental health), and (4) scientific publications excluded due to methodology (e.g., case series, case studies, case reports, reviews without methodology, letters to the editors, editorials, and comments).

### 2.2 Data management, extraction, analysis, and synthesis

**Figure 1:**
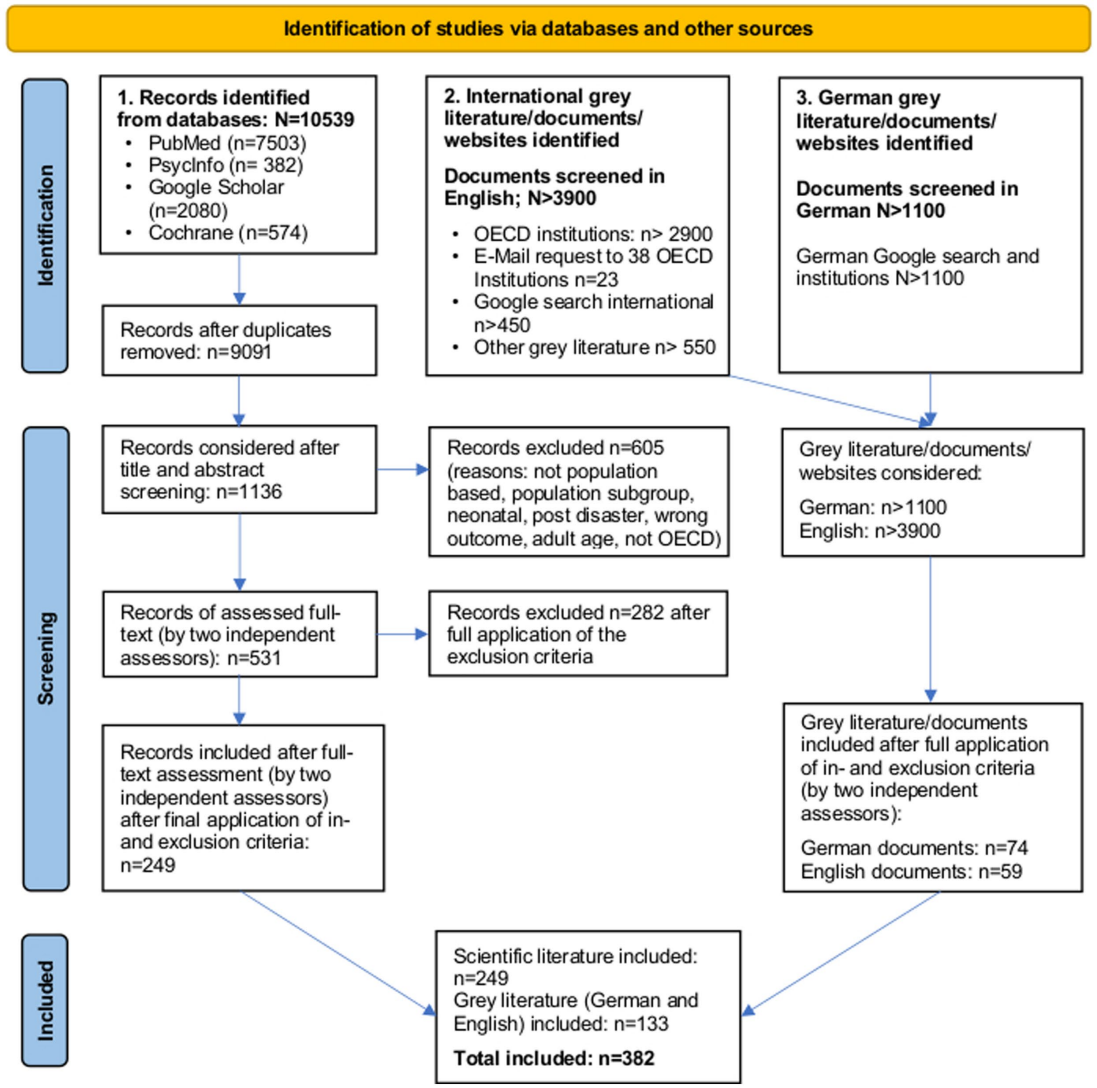
PRISMA flowchart of screening results (scientific and grey literature).

The selection process of the scientific and grey literature was conducted by three reviewers (AD, SRR, and AE), who rated each record at a different step/stage: two performed the initial selection process by title and abstract and the third reviewer made the final application of in- and exclusion criteria by full-text assessment. The abstracts of all preliminary retrieved scientific documents from the PubMed and PsycINFO search were uploaded to the software Rayyan.^6^ The records from Google Scholar and Cochrane Database were assessed online before being imported to Rayyan as the abstracts could not be imported. A duplicate check was performed automatically by the program. Any literature in which there was doubt regarding inclusion or exclusion was discussed among these three reviewers. For the screening process, no automation tool was used. Further details can be found in the PRISMA flowchart in the Results section (Figure 1).

The three reviewers screened all retrieved grey literature (international and national)/documents/websites by title and abstract or websites as appropriate. The detected websites (by one reviewer) were further screened for relevant documents, considering different layers of the website and links provided on the websites. The documents identified by two reviewers were selected in the same way described above, and those included after full-text assessment (reviewed by two reviewers) were then manually screened for duplicates. Detailed information regarding the scientific and grey literature screening process can be found in the PRISMA flowchart (Figure 1).

A data extraction form was developed by the principal investigators (AD and SRR) in an Excel sheet. It included the following predefined categories of the extracted documents: Title, reference, language, publication year, country, and study design.

Definition of indicators as used in this scoping review.

Indicators for ongoing surveillance are defined as having a clear title (1st level), a clear operationalization with explicit numerator and denominator concepts (2nd level), and the provision of an explicit database (3rd level) to compare data over time (22). To generate a broad but clear overview of additional indicators for future monitoring work in relation to children and adolescent public mental health, indicators were processed on the title level in the context of the work presented here. Therefore, theoretical concepts with empirical application at the population level are referred to as indicators, although some were not indicators in a strict sense (e.g., lacking operationalization) (23).

To describe the individual indicators, the following indicator categories were further extracted: indicator name, age range, application setting (e.g., at the national level, supranational level, or any regional level; this might be a province or any other specific area), number of measurement points over time, superordinate mental health topic, indicator type/purpose, indicator definition/operationalization, measurement tool, mode of data collection (e.g., online, face to face, and proxy), data source (e.g., questionnaire, routine data, and official statistic), and indicator evidence. Comments were added, for example, if the indicator had limited information (e.g., on operationalization or other relevant information for a critical appraisal).

Age ranges have been categorized according to former RKI work (24). We assigned to each indicator a specific age range (reflecting also our inclusion criteria of the included documents).

Following the definition of an indicator on title level within this study (see above), not only duplicates but similar concepts and expressions of an indicator (e.g., “smoking” and “tobacco use”) were grouped under the main indicator name. The frequency of how often the concept was found in the literature was counted. Some of the identified indicators have been further subcategorized if the indicator concepts were too heterogeneous (e.g., healthy lifestyle; for detailed information, see Results section).

The predefined categories (e.g., “superordinate mental health topic”) have been preselected to compare indicators and mental health topics identified in this study to those of Peitz and colleagues (20) regarding indicators for adult public mental health. This should facilitate the integration of the findings in the already established MHS for Germany at RKI and the identification of indicator concepts specific to the age group of children and adolescents, taking into account their very special life and development situations.

To be able to extract all data comprehensively in a limited time despite the large number of records, AD, SRR, and AE were supported by MO and *CF* to categorize and analyze the indicators according to the above-given categories. Additionally, indicators were checked for correct assignment (DP, JT, and EM) and the following exclusion criteria:

Indicators were excluded if.

1) They were not relevant or feasible for continued and population-based monitoring, as they were focusing on a population sub-group living with a specific disease (e.g., intellectual disabilities). In other words, with regard to feasibility in the context of population-based monitoring, we have excluded indicators that describe the mental health of an individual group with group-specific parameters, e.g., the care situation of people with intellectual disabilities, as the indicators presented in the scoping review are intended to be usable for population-based monitoring. The frequency of a specific disease in the overall population (e.g., the prevalence of intellectual disability in the general population) was not excluded *a priori* in this way.
2) They were not supportive of the mental health of children and adolescents but reflected other age groups or were unspecified with regard to age.
3) They lacked operationalization nor information on key concepts that could be measured in an MHS.

The study team (AD supported by SRR) used the software SAS for data management and cleaning. All data extraction tables were imported into SAS and merged. Extensive data cleaning was performed: In the first step, all missing values were completed in several rounds of feedback with the data extraction team (see above). Next, implausible values were cross-checked (e.g., “measurement points” of more than 50). Semi-automated tools and macros were applied to complete data cleaning.

Data analyzes were performed in SAS on (1) document level (all included documents) and (2) indicator level (all extracted and finally included indicators). Included documents were analyzed by frequency tables for certain variables as extracted in the data extraction matrix, such as the type of the documents (scientific vs. grey literature), source of the documents (international vs. German), and year of publication or study type. These results are presented in the descriptive result section and illustrated as appropriate.

For the analytical part, the indicators were assigned to 14 predefined superordinate topics, as outlined by Peitz and colleagues for indicators referring to adults (20) to consolidate duplicates and similar content, to organize and group them effectively, and to compare them to the indicators found in the adult population. When child and adolescent indicators that did not fit into any predefined superordinate topics for adult indicators were identified, a new topic was created, leading to 19 superordinate topics (see Table 1 in the Results section for further details). Furthermore, we added, for example, under the superordinate topic “*mental health promotion/prevention*,” the indicator “Mental Health Policies/Frameworks and Governance,” as the authors perceive it of interest to the international community. In that regard, the superordinate topics were expanded to include four further topics specific to that age group of children and adolescents, resulting in 19 superordinate topics to classify this study’s findings (see Table 1). DP, JT, and EM contributed to quality control during the development of superordinate topics and indicator categories by contributing their former experience in an iterative process. In Table 2, a column labeled “new” indicates whether the specific indicator was newly introduced compared to Peitz et al.’s (20) work on the adult population. This is intended to mark those indicators identified in the specific searches for children and adolescents. Another column labeled “*N*” displays the number of single identified indicator concepts with similar content and/or duplicates aggregated under each specific indicator title, i.e., shows how often the respective indicator was found. This column helps illustrate the level of usage or discussion of each indicator within the literature. To provide examples for each indicator, one or two relevant references have been selected.

**Table 1:** Nineteen superordinate topics of mental health indicators for children and adolescents, numbers of and shares of identified indicators per topic separately for international and German literature.

Classification	International		German origin/in German		*p*-value
	(*N*)	(%)	(*N*)	(%)	
Mental Health Promotion and Prevention	64	1.3	100	6.9	< 0.0001^a^
Psychological Resources	1,109	22.3	146	10.1	< 0.0001^a^
Social Resources	953	19.1	167	11.6	< 0.0001^a^
Individual Risks	669	13.4	238	16.5	0.003^a^
Social Risks	511	10.3	100	6.9	0.0001^a^
Mental Health Literacy	25	0.5	.	.	
Positive Mental Health	260	5.2	31	2.1	< 0.0001^a^
Preclinical Symptoms	16	0.3	6	0.4	0.6^a^
Mental Disorders/Psychopathology	821	16.5	347	24.0	< 0.0001^a^
Comorbidity	19	0.4	14	1.0	0.006^a^
Self-Harm/Suicidality	128	2.6	4	0.3	< 0.0001^a^
Supply and Utilization of Mental Health Care	199	4.0	204	14.1	< 0.0001^a^
Quality of Care	44	0.9	22	1.5	0.03^a^
Needs, Unmet Needs, and Barriers in Mental Health Care	30	0.6	7	0.5	0.6^a^
Costs due to Mental Disorders	12	0.2	5	0.3	0.5^a^
Burden of Disease	6	0.1	2	0.1	1.0^b^
Mortality	3	0.1	.	.	
Participation	7	0.1	1	0.1	0.7^b^
Sociodemographic Variables with an impact on public mental health	107	2.1	49	3.4	0.007^a^
Total	4,983	100	1,443	100	

a Chi-square test, two-sided.

b Fisher’s exact test, two-sided.

**Table 2:** Public mental health indicators for children and adolescents: information on the number identified per indicator, new indicator development compared to existent indicators for the adult population, current use in surveillance systems, and reporting by age groups.

	Indicator	*N*	%	Frequency of category	New	Surveillance	Age groups								International reference	German reference
							0–2	3–5	6–8	9–11	12–14	15–17	18–24	>24		
Mental Health Promotion and Prevention																
1	School Entry Examination	65	1.01	>10	x			x	x							(25)
2	Anti-Stigma Movement	1	0.02	1		Possibly									(26)	
3	Early Intervention [e.g., in Psychosis]	7	0.11	2–10		Yes				x	x	x	x		(27)	(28)
4	Existence of Mental Health Promotion Programs	12	0.19	>10		Yes	x	x	x	x	x	x	x		(27)	
5	Participation in Selected or Indicated Preventive Programs on Mental Health	4	0.06	2–10		Yes										(29)
6	Presence of Programs to Support Parenting Skills	5	0.08	2–10		Yes	x	x							(30)	(31)
7	Presence of Sexual Abuse Prevention Programs/Instruments	7	0.11	2–10	x	Yes					x	x	x		(27)	
8	Presence of Mental Health Promotion in Schools	11	0.17	>10		Possibly		x	x	x	x	x	x	x		(32)
9	Mental Health Promotion and Prevention Budget	4	0.06	2–10		Yes										(33)
10	Utilization of Health Promotion and Early Detection Programs	23	0.36	>10	x	Yes	x	x	x	x	x	x			(34)	(35)
11	Mental Health Policies/Frameworks and Governance	25	0.39	>10	x	Yes	x	x	x	x	x				(27)	(29)
Psychological Resources																
12	Optimism	20	0.31	>10		Possibly	x	x	x	x	x	x	x	x	(36)	(37)
13	General Trust	10	0.16	2–10		Yes	x	x	x	x	x	x	x		(38)	(37)
14	Self-Esteem	57	0.89	>10		Yes	x	x	x	x	x	x	x	x	(39)	
15	Self-Efficacy	26	0.40	>10		Yes	x	x	x	x	x	x	x	x	(40)	
16	Resilience	62	0.96	>10		Yes	x	x	x	x	x	x	x	x		(37)
17	Coping (e.g., Emotion Regulation/Function, Realistic Goals, Responsibility, Positive/Negative Affect)	51	0.79	>10		Yes	x	x	x	x	x	x	x		(30)	(25)
18	Emotional Relational Development	103	1.60	>10	x	Yes	x	x	x	x	x	x	x		(38)	
19	Personality	70	1.09	>10		Yes	x	x	x	x	x	x	x		(15)	(41)
20	Cognitive Development and Function	64	1.00	>10	x	Yes	x	x	x	x	x	x	x		(27)	(42)
21	Breastfeeding	10	0.16	2–10	x	Yes	x	x	x	x	x	x	x	x	(43)	(35)
22	Healthy Lifestyle [e.g., Nutrition, Physical Activity, Substance/Alcohol Consumption]	559	8.70	>10		Yes	x	x	x	x	x	x	x	x	(38)	
23	Spirituality	32	0.50	>10		Yes		x	x	x	x	x	x		(44)	
24	Sense of Coherence	2	0.03	2–10	x				x	x	x	x	x		(45)	(37)
25	Media Use	85	1.32	>10	x	Yes	x	x	x	x	x	x	x	x	(46)	
26	Self-Managed and Leisure Time (Activities)	55	0.86	>10	x	Yes	x	x	x	x	x	x	x	x	(38)	(47)
27	Sleep	49	0.76	>10		Yes	x	x	x	x	x	x	x		(48)	(49)
Social Resources																
28	Life-Domain/Work-Life Balance (Parents)	2	0.03	2–10			x	x	x	x	x	x	x	x	(36)	
29	Neighborhood Environment (Social and Build)	73	1.14	>10		Yes	x	x	x	x	x	x	x		(48)	(50)
30	Perceived Neighborhood Security	29	0.45	>10		Yes	x	x	x	x	x	x	x		(38)	(42)
31	Sense of Community Belonging	28	0.44	>10		Yes			x	x	x	x	x		(43)	
32	Community Involvement (Self/Parents)	34	0.53	>10		Yes	x	x	x	x	x	x	x	x	(51)	(52)
33	Political Participation	12	0.19	>10		Yes			x	x	x	x	x		(38)	
34	Social Support/Social Network	235	3.66	>10		Yes	x	x	x	x	x	x	x	x	(38)	(40)
35	Access to Educational Resources	15	0.23	>10	x	Yes	x	x	x	x	x	x	x	x	(53)	
36	School-Related Resources (Environment, Satisfaction, Support, Motivation, Achievement)	331	5.15	>10	x	Yes		x	x	x	x	x	x	x	(38)	(37)
37	Adherence to Social and Cultural Norms	25	0.39	>10	x	Yes		x	x	x	x	x	x		(38)	(50)
38	Availability of Public Transportation	2	0.03	2–10	x										(54)	
39	Community Environment/Connectedness	5	0.08	2–10	x	Possibly		x	x	x	x	x	x	x	(55)	
40	Family Functionality	148	2.30	>10		Yes	x	x	x	x	x	x	x	x	(27)	(56)
41	Family Wellbeing [e.g., Emotional/Subjective Well-Being; Psychological, Social, Physical]	23	0.36	>10	x	Yes	x	x	x	x	x	x	x		(38)	
42	Parenting Style and Skills	157	2.44	>10		Yes	x	x	x	x	x	x	x	x	(30)	(31)
43	Teachers/Other Responsible Adults Wellbeing [e.g., Emotional/Subjective Wellbeing; Psychological, Social, Physical]	1	0.02	1	x				x	x	x	x				(40)
Individual Risks																
44	Adverse Childhood Experiences and Trauma (e.g., Sexual, Physical, and Emotional Abuse; Parental Criminality)	229	3.56	>10		Yes	x	x	x	x	x	x	x	x	(30)	(28)
45	Critical Life Events	10	0.16	2–10			x	x	x	x	x	x	x	x	(57)	
46	Bullying	75	1.17	>10	x	Yes	x	x	x	x	x	x	x		(38)	
47	Discrimination	14	0.22	>10		Yes	x	x	x	x	x	x	x		(38)	
48	Violence	9	0.14	2–10		Yes	x	x	x	x	x	x	x		(58)	
49	Chronic Pain	4	0.06	2–10			x	x	x	x	x	x			(59)	
50	Chronic Physical Condition and Physical Health Problems	346	5.38	>10	x	Yes	x	x	x	x	x	x	x	x	(38)	(42)
51	Chronic Stress	11	0.17	>10		Possibly	x	x	x	x	x	x	x		(60)	
52	Family History of Mental Disorders and Mental Health Problems	73	1.14	>10		Yes	x	x	x	x	x	x	x		(61)	
53	Family History of Suicide-Related Behavior	4	0.06	2–10			x	x	x	x	x				(62)	
54	Perinatal and Antenatal Background	77	1.20	>10	x	Yes	x	x	x	x	x	x	x	x	(27)	(37)
55	Special Needs	10	0.16	2–10	x	Yes	x	x	x	x	x	x	x		(30)	(33)
56	Teen Pregnancy (Self/Parents)/STI	45	0.70	>10	x	Yes	x	x	x	x	x	x	x	x	(30)	(40)
Social Risks																
57	Loneliness	20	0.31	>10		Yes		x	x	x	x	x	x		(38)	(63)
58	Risky Behavior of Peers/Friends	3	0.05	2–10	x	Yes				x	x	x	x		(48)	
59	Availability of/Access to Weapons	1	0.02	1	x	Yes										(50)
60	School-Related Risks (Problems/Attendance/Readiness/Strain)	96	1.49	>10	x	Yes			x	x	x	x	x	x	(38)	(64)
61	Language Barriers	4	0.06	2–10	x			x	x	x	x	x	x		(36)	(65)
62	Cognitive Impairment	14	0.22	>10		Yes	x	x	x	x	x	x	x		(30)	(66)
63	Chronic Stress in the Family	7	0.11	2–10	x	Yes	x	x	x	x	x	x	x		(67)	(31)
64	Chronically Ill Parent	6	0.09	2–10	x	Yes	x	x	x	x	x	x	x		(48)	(68)
65	Parental Risk Behavior	3	0.05	2–10	x											(69)
66	Household Composition and Family Structure	99	1.54	>10	x	Yes	x	x	x	x	x	x	x	x	(30)	
67	Family Socio-Economic Situation (Employment Status, Household Income, etc.)	254	3.95	>10	x	Yes	x	x	x	x	x	x	x	x	(30)	
68	Problematic Housing Conditions	61	0.95	>10		Yes	x	x	x	x	x	x	x	x	(48)	(50)
69	Stressful Neighborhood Conditions	9	0.14	2–10		Yes	x	x	x	x	x	x	x		(70)	(35)
70	Homelessness	5	0.08	2–10		Yes						x	x		(38)	
71	Income Equality/Social Deprivation of the District (GINI)	23	0.36	>10		Yes	x	x	x	x	x	x	x		(38)	(71)
72	Income Inequality in Society	4	0.06	2–10		Yes	x	x	x	x	x	x	x		(38)	
73	Climate Change	2	0.03	2–10	x	Yes	x	x	x	x	x	x			(48)	(72)
Mental Health Literacy																
74	Mental Health-Related Knowledge	6	0.09	2–10		Possibly				x	x	x	x	x	(73)	
75	Mental Health Locus of Control	11	0.17	>10		Yes	x	x	x	x	x	x	x		(15)	
76	Help-Seeking Attitudes and Behavior	8	0.12	2–10		Possibly		x	x	x	x	x	x		(74)	
Positive Mental Health																
77	Happiness	30	0.47	>10		Yes				x	x	x	x		(38)	
78	Health-Related Quality of Life	67	1.04	>10		Yes	x	x	x	x	x	x	x	x	(75)	(47)
79	Life Satisfaction	83	1.29	>10		Yes		x	x	x	x	x	x	x	(41)	
80	Wellbeing	100	1.56	>10		Yes	x	x	x	x	x	x	x	x	(38)	(49)
81	Meaning in Life	11	0.17	>10		Yes				x	x	x	x		(48)	(37)
Preclinical Symptoms																
82	Prevalence of Psychological Distress	22	0.34	>10		Yes	x	x	x	x	x	x	x	x	(76)	(49)
Mental Disorders/Psychopathology																
83	General Mental Health Status	49	0.76	>10	x	Yes	x	x	x	x	x	x	x		(43)	(42)
84	Incidence of Anxiety Disorders	7	0.11	2–10			x	x	x	x	x	x	x		(77)	
85	Incidence of Any Mental Disorders	3	0.05	2–10			x	x	x	x	x	x	x	x	(78)	
86	Developmental Delay and Disorders	84	1.31	>10	x	Yes	x	x	x	x	x	x	x		(30)	(28)
87	Prevalence of Adjustment Disorder	6	0.09	2–10			x	x	x	x	x	x	x		(79)	(80)
88	Prevalence of Anxiety Disorders	70	1.09	>10		Yes	x	x	x	x	x	x	x	x	(30)	(81)
89	Prevalence of Attention Deficit Disorders (ADD) and Attention Deficit Hyperactivity Disorders (ADHD)	85	1.32	>10		Yes	x	x	x	x	x	x	x		(38)	(80)
90	Prevalence of Autism	20	0.31	>10	x	Yes	x	x	x	x	x	x	x		(82)	
91	Prevalence of Bipolar Disorders	6	0.09	2–10				x	x	x	x	x	x		(83)	
92	Prevalence of Conduct Disorder	138	2.15	>10	x	Yes	x	x	x	x	x	x	x		(38)	(84)
93	Prevalence of Depression and/or Anxiety Disorders	16	0.25	>10		Yes	x	x	x	x	x	x	x		(30)	
94	Prevalence of Eating Disorders	25	0.39	>10		Yes	x	x	x	x	x	x	x		(38)	
95	Prevalence of Impulse Control Disorders	1	0.02	1			x	x	x	x	x	x	x			(80)
96	Prevalence of Manic Episodes	5	0.08	2–10				x	x	x	x	x	x			(80)
97	Prevalence of Obsessive-Compulsive Disorder	11	0.17	>10				x	x	x	x	x	x		(85)	(80)
98	Prevalence of Personality Disorders	16	0.25	>10			x	x	x	x	x	x	x		(86)	(80)
99	Prevalence of Posttraumatic Stress Disorder	4	0.06	2–10						x	x	x	x		(86)	
100	Prevalence of Psychotic Disorder	14	0.22	>10		Yes	x	x	x	x	x	x	x		(30)	(87)
101	Prevalence of Schizophrenia	9	0.14	2–10		Yes	x	x	x	x	x	x	x		(82)	(80)
102	Prevalence of Sexual Dysfunctions	2	0.03	2–10	x							x	x		(88)	(80)
103	Prevalence of Sleep Disorders	4	0.06	2–10			x	x	x	x	x	x	x		(89)	(80)
104	Prevalence of Somatoform and Dissociative Disorders	17	0.26	>10			x	x	x	x	x	x	x		(90)	(28)
105	Prevalence of Substance Use Disorder	21	0.33	>10		Yes	x	x	x	x	x	x	x	x	(30)	(68)
106	Prevalence of Internalization Problems	40	0.62	>10	x	Possibly	x	x	x	x	x	x	x		(91)	(64)
107	Prevalence of Externalization Problems	106	1.65	>10	x	Yes	x	x	x	x	x	x	x	x	(92)	
108	Presence of Mental Health Problems (not Specified)	228	3.55	>10	x	Yes	x	x	x	x	x	x	x	x	(30)	(93)
109	Prevalence of Chronic Mental Disorders	5	0.08	2–10			x	x	x	x	x	x	x	x	(94)	(95)
110	Prevalence of Mood/Affective Disorders or Depression	122	1.90	>10		Yes	x	x	x	x	x	x	x	x		(80)
111	Prevalence of any Mental Disorder (all F-Diagnoses)	54	0.84	>10		Yes	x	x	x	x	x	x	x	x	(78)	
Comorbidity																
112	Comorbidity Physical Disease	31	0.48	>10		Yes	x	x	x	x	x	x	x		(96)	
113	Comorbidity Mental Disorder	2	0.03	2–10				x	x	x	x				(62)	(87)
Self-Harm and Suicidality																
114	Self-Harm	34	0.53	>10		Yes	x	x	x	x	x	x	x		(30)	(63)
115	Suicidality [e.g., Ideations, Plans]	49	0.76	>10		Yes				x	x	x	x		(30)	(69)
116	Suicide Attempts	18	0.28	>10		Yes		x	x	x	x	x	x		(30)	
117	Suicide Rate	31	0.48	>10		Yes	x	x	x	x	x	x	x	x	(82)	
Supply and Utilization of Mental Health Care																
118	Outpatient: Capacity of Child and Youth Mental Health Care, Mental Health Workers	12	0.19	>10		Yes				x	x	x	x		(27)	(97)
119	Outpatient: Capacity of Child and Youth Mental Health Care, Mental Health Specialists	30	0.47	>10		Yes	x	x	x	x	x	x	x		(98)	(99)
120	Inpatient: Capacity of Child and Youth Psychiatry, Human Resources	5	0.08	2–10		Yes	x	x	x	x	x	x	x		(82)	(97)
121	Inpatient: Capacity of Child and Youth Psychiatry, Treatment and Services	10	0.16	2–10	x	Yes	x	x	x	x	x	x	x		(82)	(100)
122	Inpatient: Capacity of child and youth psychiatry, number of beds	12	0.19	>10	x	Yes	x	x	x	x	x	x	x			(32)
123	Inpatient: Capacity of Child and Youth Psychiatry, not Specified/Other	10	0.16	2–10	x	Yes	x	x	x	x	x	x	x			(101)
124	General: Coverage of Services for Severe Mental Disorders	1	0.02	1		Possibly									(102)	
125	General: Treatment Coverage for Alcohol and Drug Dependence	2	0.03	2–10		Yes	x	x	x	x	x	x	x		(82)	
126	General: Treatment with Psychotropic Drugs	3	0.05	2–10			x	x	x	x	x	x	x		(103)	
127	General: Reasons for Treatment	3	0.05	2–10	x						x	x	x		(104)	(100)
128	General: Utilization of Any Healthcare of Children and Youth with Diagnosed Mental Disorders	4	0.06	2–10		Yes					x	x			(105)	
129	Inpatient: Number of Child and Youth Psychiatric Hospitals	3	0.05	2–10		Yes	x	x	x	x	x	x	x			(99)
130	Inpatient: Number of Psychiatric Units for Youth and Children in General Hospitals	2	0.03	2–10		Yes	x	x	x	x	x	x	x			(99)
131	Inpatient: Number of Forensic Units	1	0.02	1		Possibly									(106)	
132	Outpatient: Number of Mental Health Facilities for Children and Adolescents Attached to a Hospital	2	0.03	2–10		Yes	x	x	x	x	x	x	x			(99)
133	Inpatient: Number of Cases	7	0.11	2–10		Yes	x	x	x	x	x	x	x		(82)	(99)
134	Inpatient: Number of Days of Stay	20	0.31	>10		Yes	x	x	x	x	x	x	x		(82)	(99)
135	Inpatient: Number of Long Stay Patients	3	0.05	2–10												(100)
136	Inpatient: Readmissions by Mental Health Diagnoses	7	0.11	2–10		Yes	x	x	x	x	x	x	x		(82)	
137	Inpatient: Utilization of Care of Children and Youth with Diagnosed Mental Disorders	42	0.65	>10		Yes	x	x	x	x	x	x	x	x	(27)	(101)
138	Inpatient: Utilization of Child and Youth Psychiatry: Treatment and Services	7	0.11	2–10	x	Yes	x	x	x	x	x	x	x		(89)	(64)
139	Others: Capacity of Child and Youth Mental Health Services, not Specified/Other	10	0.16	2–10	x		x	x	x	x	x	x				(101)
140	Others: Capacity of Children and Youth Mental Health Consultation Services (Crisis Line, etc…)	3	0.05	2–10	x										(107)	
141	Others: Capacity of Outpatient Youth Welfare (Jugendhilfe)	3	0.05	2–10	x				x	x	x	x	x			(64)
142	Others: Capacity of Social Pediatric Centers (SPZ), not Specified	4	0.06	2–10	x											(32)
143	Others: Capacity of Support for Education/Upbringing	1	0.02	1	x											(32)
144	Others: Children and Youth Protection Programs/Measures, not Specified/Other	22	0.34	>10	x	Yes	x	x	x	x	x	x	x		(30)	
145	Others: Emergency Care	10	0.16	2–10		Yes	x	x	x	x	x	x	x		(27)	
146	Others: Opioid Substitution Treatment	3	0.05	2–10				x	x	x	x	x			(89)	
147	Others: Self-Help Intervention Capacity	1	0.02	1	x											(29)
148	Others: Utilization of Foster Care	11	0.17	>10	x	Yes	x	x	x	x	x	x	x		(27)	
149	Others: Utilization of Outpatient Children and Youth Mental Health Consultation Services (Crisis Line, etc…)	10	0.16	2–10	x	Yes	x	x	x	x	x	x	x		(82)	
150	Others: Utilization of Outpatient Youth Welfare (Jugendhilfe)	6	0.09	2–10	x		x	x	x	x	x	x	x	x		(64)
151	Others: Utilization of Public Assistance for Children and Youth (e.g., Financial Support)	10	0.16	2–10	x	Yes	x	x	x	x	x	x	x	x	(27)	(52)
152	Others: Utilization of Rehabilitation Measures due to Mental Disorders	9	0.14	2–10		Yes						x	x		(108)	(109)
153	Others: Utilization of Social Pediatric Centers (SPZ)	1	0.02	1	x		x	x	x	x	x	x	x	x		(32)
154	Others: Utilization of Stationary Youth Welfare (Jugendhilfe)	2	0.03	2–10	x											(97)
155	Others: Utilization of Support Services After Sexual Abuse	11	0.17	>10	x		x	x	x	x	x	x				(25)
156	Others: Utilization of Support for Education/Upbringing	1	0.02	1	x											(32)
157	Outpatient: Aftercare	3	0.05	2–10											(62)	(32)
158	Outpatient: Capacity of Child and Youth Mental Health Care, Treatment and Services	4	0.06	2–10	x	Yes	x	x	x	x	x	x	x		(67)	(99)
159	Outpatient: Capacity of Child and Youth Psychiatric Day Care	6	0.09	2–10	x	Yes	x	x	x	x	x	x				(101)
160	Outpatient: Proportion of Pharmacotherapy in Mental Health Care	4	0.06	2–10		Yes	x	x	x	x	x	x	x		(30)	(110)
161	Outpatient: Treatment of Children and Youth with Psycho- and Pharmacotherapy	35	0.54	>10		Yes	x	x	x	x	x	x	x	x	(43)	(64)
162	Outpatient: Utilization of Child and Youth Mental Health Care: Treatment and Services	26	0.40	>10	x	Yes	x	x	x	x	x	x	x		(30)	
163	Outpatient: Utilization of Child and Youth Psychiatric Day Care	2	0.03	2–10			x	x	x	x	x	x	x			(111)
164	Outpatient: Utilization of Home Treatment	11	0.17	>10		Yes	x	x	x		x	x	x		(27)	
165	Outpatient: Utilization of Mental Health Care of children and youth with diagnosed mental disorders	3	0.05	2–10		Yes	x	x	x	x	x	x	x		(30)	(112)
166	Outpatient: Utilization of Primary Healthcare of Children and Youth with Diagnosed Mental Disorders for Mental Health Reasons	4	0.06	2–10	x	Yes	x	x	x	x	x	x	x		(30)	
167	Outpatient: Utilization of Primary Healthcare of Children and Youth with Diagnosed Mental Disorders for Physical Health Reasons	1	0.02	1	x					x	x				(113)	
Quality of Care																
168	Patient/Parents or Family Satisfaction with Mental Health Care Services	12	0.19	>10		Yes	x	x	x	x	x	x	x			(72)
169	Patient Reported Outcome Measures	1	0.02	1							x	x	x		(104)	
170	Treatment Success	3	0.05	2–10								x	x	x		(32)
171	Patient Education and Participation	24	0.37	>10		Possibly			x	x	x	x	x	x	(114)	(32)
172	Inclusion of Family and Social Environment into Treatment of Mental Disorders	1	0.02	1											(115)	
173	Coercive Measures: Compulsory Treatment	9	0.14	2–10			x	x	x	x	x	x	x		(108)	(63)
174	Coercive Measures: Seclusion	4	0.06	2–10		Yes									(62)	
175	Coercive Measures: Fixation	2	0.03	2–10	x											(32)
176	Outpatient-sensitive hospital cases	1	0.02	1			x	x	x	x	x				(116)	
177	National Standards for Health Service Delivery for Young People	1	0.02	1	x	Possibly									(114)	
178	Quality of Mental Health Services	8	0.12	2–10	x	Yes	x	x	x	x	x	x	x		(27)	
Needs, Unmet Needs, and Barriers in Mental Health Care																
179	Perceived Needs	4	0.06	2–10			x	x	x	x	x	x	x	x	(117)	(52)
180	Treatment Latency	1	0.02	1		Possibly									(102)	
181	Met Mental Health Care Needs for Children/Adolescents with Mental Disorders	6	0.09	2–10	x	Yes					x	x	x		(30)	
182	Unmet Mental Health Care Needs	2	0.03	2–10		Possibly		x	x	x	x	x	x		(118)	
183	Waiting Times for Mental Health Care Services	5	0.08	2–10		Yes	x	x	x	x	x	x	x		(27)	(25)
184	Access Barriers in Mental Health Care	5	0.08	2–10		Yes		x	x	x	x	x			(27)	
185	Existence of Coordination Measures for the Management of Mental Disorders	7	0.11	2–10	x	Possibly	x	x	x	x	x	x			(119)	(29)
186	Transfer Rate from Primary to Secondary Care	7	0.11	2–10		Yes	x	x	x	x	x	x	x		(30)	
Costs due to Mental Disorders																
187	Direct Costs due to Mental Disorders	1	0.02	1												(120)
188	Direct Costs due to Mental Disorders—Outpatient Care	1	0.02	1												(121)
189	Direct Costs due to Mental Disorders—Rehabilitation	6	0.09	2–10			x	x	x	x	x	x	x	x	(108)	(120)
190	Indirect Costs due to Mental Disorders (Other)	2	0.03	2–10			x	x	x	x	x	x	x	x	(89)	
191	Indirect Costs: Disability Pension for Mental Health Reasons	2	0.03	2–10								x	x			(120)
192	Indirect Costs: Sickness Compensation	5	0.08	2–10			x	x	x	x	x	x	x		(108)	(120)
Burden of Disease																
193	Functional Impairment due to Mental Health Reasons	3	0.05	2–10		Possibly		x	x	x	x	x	x		(118)	(34)
194	Mentally Unhealthy Days	2	0.03	2–10		Yes					x	x	x		(122)	
195	DALYs (Disability-Adjusted Life Years)	3	0.05	2–10		Possibly				x	x	x	x		(123)	
Mortality																
196	Alcohol-Related Deaths	2	0.03	2–10								x	x	x	(124)	
197	Drug-Related Deaths	1	0.02	1								x	x	x	(89)	
Participation																
198	Proportion of People in Probation/Prison/Provincial Correctional Centers with Mental Illnesses	5	0.08	2–10		Yes					x	x	x			(32)
199	Social and Political Participation in People with Mental Illnesses	1	0.02	1							x	x			(125)	
200	Discrimination/Stigmatization due to Mental Health Problems	2	0.03	2–10		Yes									(38)	
Sociodemographic variables with an impact on public mental health																
201	Age	15	0.23	>10		Possibly	x	x	x	x	x	x	x	x	(126)	(84)
202	Gender	20	0.31	>10		Possibly	x	x	x	x	x	x	x		(126)	(84)
203	Region	1	0.02	1											(127)	(29)
204	Urbanization/Region	20	0.31	>10		Possibly	x	x	x	x	x	x	x		(127)	(29)
205	Migration Background/Ethnicity	41	0.64	>10		Yes	x	x	x	x	x	x	x		(48)	(84)
206	Youth Unemployment (NEET)	13	0.20	>10		Yes					x	x	x	x	(43)	(100)
207	Employment Status Youth	4	0.06	2–10	x	Possibly			x	x	x	x	x		(128)	
208	Child’s/Adolescent’s Level of Education	20	0.31	>10		Yes			x	x	x	x	x		(38)	(84)
209	Socio-Economic Status Child/Adolescent	19	0.30	>10		Possibly	x	x	x	x	x	x	x		(129)	(112)
210	Marital Status of Adolescents/Youth	3	0.05	2–10		Possibly						(x)**	x		(130)	

* “in some studies, children/adolescents, respectively their parents, were asked at certain ages whether they had been breastfed.” **in Germany, marriage at the age of 16 is allowed if the parents give consent.”

### 2.3 Quality assessment

The selected literature in this scoping review was not graded, nor was a risk of bias assessment performed due to the scoping review approach and the amount and type of literature.

## 3 Results

### 3.1 Results of the literature search

The initial scientific literature search, covering 38 OECD countries, identified *N* = 10,539 articles, including duplicates (see Figure 1). Of those, *N =* 9,091 were screened by title and abstract (if available), *N =* 1,136 articles were considered for full-text assessment as described in the methodology section, *N =* 531 were full-text assessed, and *N =* 249 articles were finally included.

Of all retrieved international grey literature/documents/websites (*N* > 3,900), *N* = 59 documents were included after the exclusion of *N* = 1 duplicate.

The search for German institutions and lay organizations yielded *N* > 1,100 grey literature/documents/websites, of which *N* = 74 documents were included after full-text assessment. In both (international and national) searches, we provided only a minimum number for the Google searches in the flowchart. This is because one initial website often contained additional sub-websites and further links, which were screened but not counted in detail. For details, see the PRISMA flowchart (Figure 1).

### 3.2 Results of included literature

**Figure 2:**
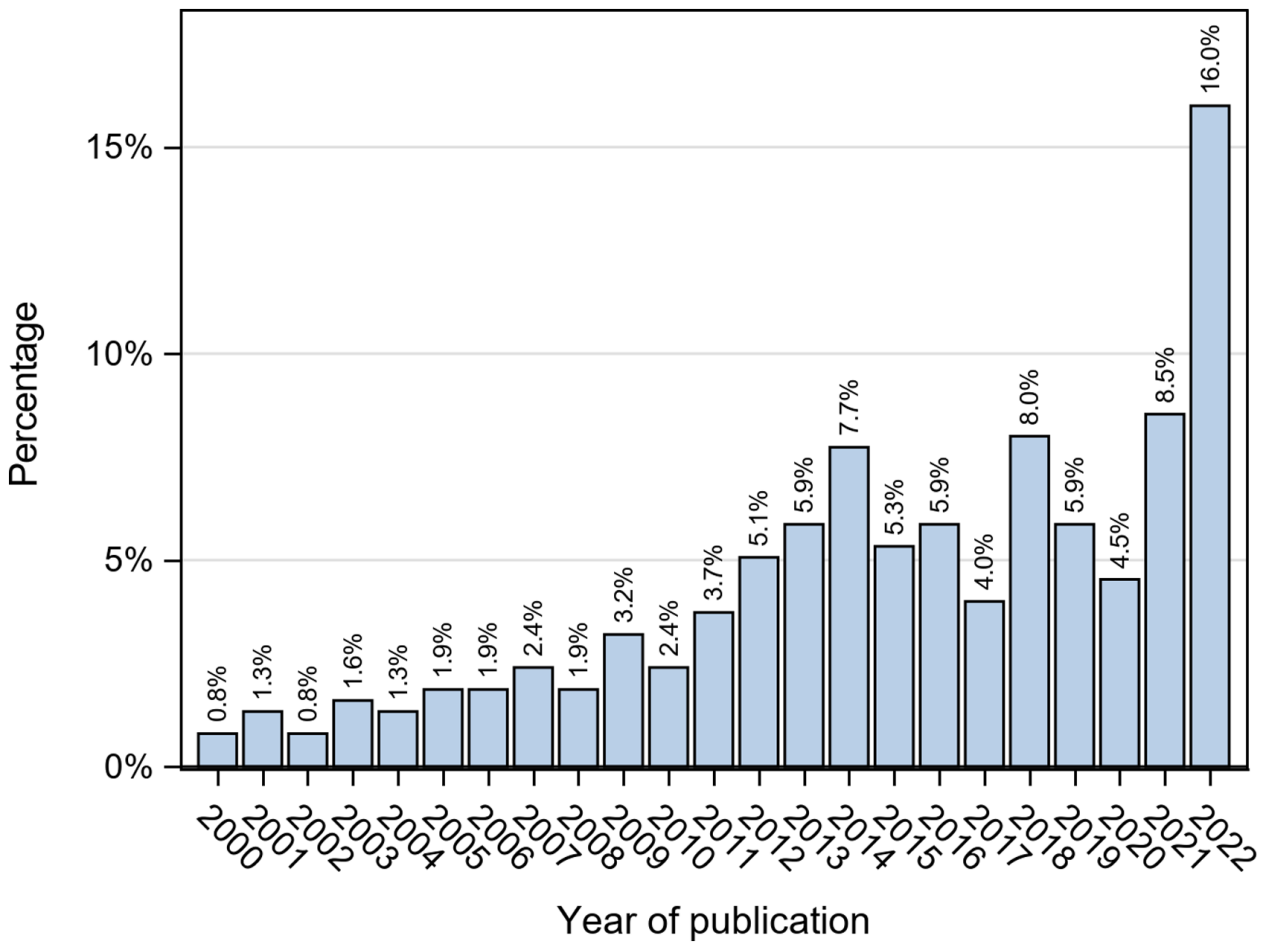
Publication year of included literature.

This study included articles/documents from 01.01.2000 to 31.10.2022, as described in the methodology and illustrated in Figure 2. The number of publications increased overall since 2008, with a peak in 2022.

The *N* = 382 included articles/documents showed the following methodological characteristics: *N* = 168 (44%) articles/documents reported results of either cross-sectional studies or surveys, *N =* 66 (17.3%) were based on longitudinal studies or data sets, *N =* 33 (8.6%) were non-specific or used mixed methods in their design, *N =* 32 (8.4%) reported about surveillance systems in use, *N =* 27 (7.1%) were cohort studies, *N =* 20 (5.2%) were reports (mostly with a non-specific study methodology), and *N =* 1 (0.3%) was a randomized-control trial. The final 9.1% of articles/documents were mostly case–control designs, document reviews or reports with a specified methodology, retrospective studies, or systematic reviews.

Off the *N =* 249 included articles of the scientific search (*N =* 2,909 initially identified indicators respectively), *N =* 5 (26 indicators) were articles identified in German language, *N =* 3 (4 indicators) were in Spanish, and *N =* 241 (*N =* 2,879 indicators) were in English.

Off the *N =* 133 included grey literature documents (*N =* 3,517 indicators respectively), *N =* 80 documents (*N =* 1,601 indicators) originated from German grey literature and *N =* 53 documents (*N =* 1916 indicators) from international (English) grey literature.

### 3.3 Results of included indicators

In total, *N* = 7,477 indicators were initially extracted from the scientific and grey literature searches. These indicators have been further screened and analyzed for (1) duplicates and similar content to be grouped under, (2) inappropriate content, (3) non-specification for mental health surveillance, or (4) indicators without clear concept or operationalization (see Methods section).

In the first step, *N* = 1,051 of the identified indicators were excluded due to reasons (2), (3), and (4), resulting in an initial set of *n* = 6,426 extracted indicators for the child and adolescent population (still including duplicates and similar content).

Of the total number of indicators identified by the scientific search (*N =* 2,909, 45.3%), most indicators were provided by documents from the United States (*N* = 778), followed by Spain (*N =* 534), Germany (*N =* 489), Canada (*N =* 483), Australia (*N =* 400), and Italy (*N =* 389). Of the other OECD countries, seven contributed with *N =* 300–399 indicators each, 16 *N =* 200–299 each, and nine *N =* 100–199 each. A further *N =* 166 indicators could not be allocated to a country. The sum of the number of indicators per country exceeds the total number of identified indicators as several indicators covered more than one country; this also applies to the following paragraph on grey literature.

Of the total number of indicators identified by the grey literature search (*N =* 3,517, 54.7%), most indicators were provided by documents from Germany (*N =* 1938), followed by the United States (*N =* 1,040), United Kingdom (*N =* 804), Canada (*N =* 789), Switzerland (*N =* 671), Finland (*N =* 633), and New Zealand (*N =* 613). All other OECD countries contributed *N =* 578–524 indicators each by this search, while *N =* 22 indicators could not be allocated to a country. Of the total amount of *N =* 6,426 indicators, *N =* 3,423 (53.3%) were applied at the national level, *N =* 1,012 (15.7%) at the supranational level, and *N =* 1,594 (24.8%) at any regional level.

From this initial set, *N =* 3,594 (55.9%) indicators were measured or mentioned once only, *N =* 1,026 (16%) indicators were measured 2–10 times, *N =* 163 (2.5%) indicators were measured more than 10 times, and *N =* 76 (1.2%) were measured regularly. For *N =* 1,567 (24%) indicators, the measuring points have not been stated.

Most indicators were retrieved by questionnaires or survey data (*N =* 358, 47.6%), followed by an integration of multiple sources (*N =* 1782, 27.7%). The others originated from health insurance data, clinical data or hospital statistics, medical birth registries, and statistic sources such as the causes of death, youth welfare offices, or others. A total of *N =* 678 (10.5%) could not be further specified in terms of this classification.

From this initial set, *N =* 815 (12.7%) indicators were already in use for surveillance purposes, and additional *N =* 881 (13.7%) were mentioned or developed for surveillance. For the latter, it was not clear if they had been applied previously.

**Figure 3:**
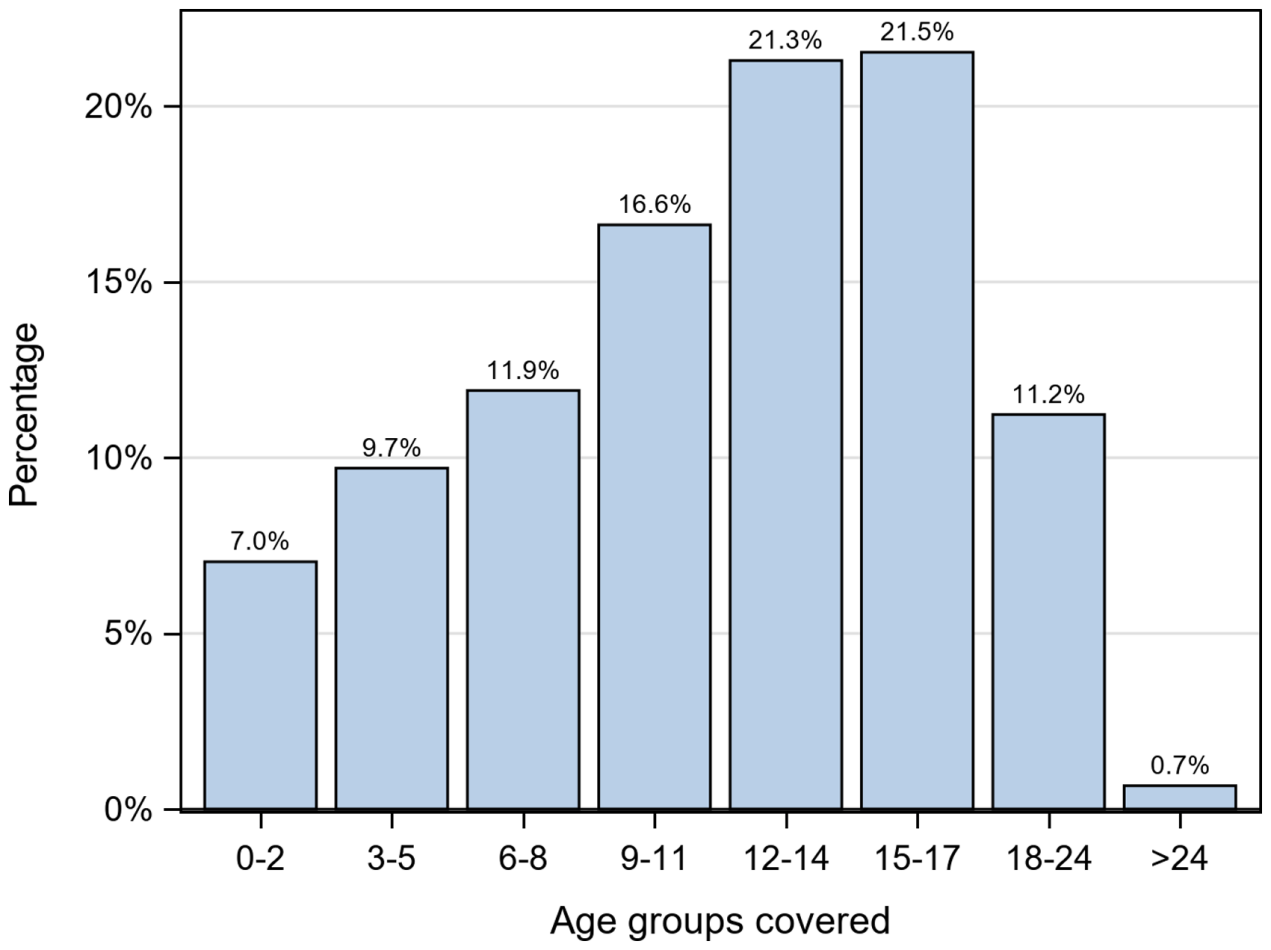
Share of age-specific reported results for identified indicators per age group.

Only 7% of the indicators covered the age group of 0–2 years in the reporting of results, whereas the highest proportion of indicators (21.5%) was reported in the age group of 15–17 years. Figure 3 illustrates the distribution of identified indicators across different age groups.

The final indicator set following this methodology included 210 different indicators. These indicators have been further categorized into two groups: those identified by the international literature and those identified through the search for German national documents.

On the level of the superordinate topics, most of the above-illustrated relative numbers and distributions differed significantly between Germany and other OECD countries. For example, in Germany, based on the relative number of the included indicators, the topics ‘*psychological resources*’ (International 22.3%; Germany 10.1%), ‘*social resources’* (International 19.1%; Germany 11.6%), “*social risks”* (International 10.3%; Germany 6.9%), ‘*positive mental health’* (International 5.2%; Germany 2.1%), and “*self-harm/suicidality”* (International 2.5%; Germany 0.3%) were represented less frequently when compared to the international literature. In contrast, the topics “*mental health promotion/prevention”* (International 1.3%; Germany 6.9%), “*mental disorder/psychopathology”* (International 16.5%; Germany 24.0%), and especially “*supply and utilization*” (International 4.0%; Germany 14.1%) were found to be represented more frequently in the German literature based on the included indicators.

The 19 superordinate topics included *N =* 210 indicators in total (see Table 1 for an overview).

Some identified indicators grouped under the respective superordinate topic have been further subcategorized. For example, under the superordinate topic ‘*individual risk*’ the indicator “Adverse Childhood Experiences & Trauma” includes indicator concepts such as, e.g., abuse and maltreatment (sexual, domestic, or emotional), victim of crime, violence, trauma, parental imprisonment, homicide, parental or sibling death, hunger or divorce within others. The indicator “Healthy Lifestyle” under the superordinate topic “*psychological resources*” includes concepts such as nutrition, diet, weight, physical or sedentary activity, and healthy regulation of substance and alcohol consumption or smoking in consensus with the standard operationalization of RKI (131). On the other hand, superordinate topics such as “*mental disorders*” offered room for overlap, specifically the indicators “Presence of Internalization Problem” and “Presence of Externalization Problems” were difficult to disentangle from “Presence of Conduct Disorder” or the “Diagnosis of Attention-Deficit/Hyperactivity Disorder (ADHD).” Despite this overlap, those indicator categories were used as they reflect more adequately the indicator terms actually identified in the selected documents.

In total, *N* = 71 new indicators could be identified. New indicators found more than 50 times in the literature were assigned to the following superordinate mental health topics: “*mental health promotion and prevention*”: (“School Entry Examination”), “*individual risk*” (“Bullying,” “Chronic Physical Condition and Physical Health Problems,” and “Perinatal and Antenatal Background”), “*psychological resources”* (“Emotional Relational Development,” “Cognitive Development and Function,” “Media Use,” and “Self-managed and Leisure Time (Activities)),” ‘*social resources*’ (“School-related Resources,” “Family Socio-economic Situation,” “Household Composition and Family Structure,” and “School-related Risks”), and “*mental disorders/psychopathology*” (“Developmental Delay and Disorders,” “Prevalence of Conduct Disorder,” “Prevalence of Externalization Problems,” and “Presence of Mental Health Problems”).

## 4 Discussion

This scoping review builds largely upon the previous study by Peitz et al. (20), which focused on indicators monitoring adult mental health at the population level. The current study aims to identify indicators specifically tailored to children and adolescents, considering their age-specific risks and resources related to mental health. It is pioneering work and, therefore, lacks references that could be used for comparison for the further identification of gaps. To address the research questions of our study, we conducted a comprehensive scoping review that encompassed scientific and grey literature from 38 OECD countries. Initially screening over 15,562 documents, we identified 6,426 indicators that met our study’s inclusion criteria and were subsequently included in the final data analysis to arrive at a final set of 210 indicator categories. The subsequent paragraphs of the discussion section will begin by (1) providing a broad overview of our results, followed by an analysis of how they relate to the research questions outlined in the introduction, including (2) indicators on the mental health of children and adolescents for application in public health surveillance, the current state in Germany and other OECD countries, and (3) current scientific gaps in reporting children’s and adolescents’ mental health reflected by unattended domains.

Table 1 presents the indicators identified across the 38 OECD countries. These indicators were named based on the indicator used in Peitz et al.’s (20) previously cited work on adult indicators. However, certain modifications were made to adapt them to the population subgroup of children and adolescents. For instance, the indicator “Patient Satisfaction with Mental Health Care System” was renamed as “Patient/Parents or Family Satisfaction with Mental Health Care Services,” and “Capacity of Outpatient Mental Health Care: Mental Health Specialists” was changed to “Capacity of Outpatient Child and Adolescents Mental Health Care: Mental Health Specialists.” Similarly, “Number of Mental Health Hospitals” was revised to “Number of Child and Youth Psychiatric Hospitals.”

In comparing the identified indicators with those from the previous study on adult mental health monitoring, our analysis revealed the emergence of 71 new indicators, as presented in Table 1. These newly identified concepts primarily focus on the mental health of children and adolescents and their very special life and development situations. Therefore, they encompass aspects such as “School Entry Examination,” “Bullying,” “Peri- and antenatal Background,” “Teen Pregnancies,” “Breastfeeding,” and “Access to Educational Resources,” among others. However, certain indicators identified in this review but not in those by Peitz et al. of the adult population have emerged to be not specific for the age group of children and adolescents, such as “Language Barriers,” “Stressful Neighborhood Conditions,” “Climate Change,” “Prevalence of Sexual Dysfunction,” and “Existence of Coordination Measures for the Management of Mental Disorders.”

Conversely, the study by Peitz et al. (20) identified indicators that were not identified in our review. For instance, under the superordinate topic of “*mental health literacy”*, their scoping review included indicators such as “Attitudes towards Mental Health,” “Attitudes towards Mental Health Services,” “Self-Stigma,” and “Social Distance towards Persons with Mental Disorders,” all aspects of mental health literacy. These indicator categories, for example, were not specifically identified in our analysis, indicating that these areas have not yet played a role in monitoring children and adolescents’ mental health.

In some cases, indicators were found that overlapped. This is a result of the different usages and definitions in different studies depending on the tradition and the purpose of the respective studies. To reflect the state of research, we have documented accordingly.

By uncovering both indicators identified in the previous review study and new indicators specific to children and adolescents, the present study provides a comprehensive understanding of the current state of mental health indicators for this age group. These findings contribute to the development of effective public health surveillance strategies for children and adolescents.

### 4.1 Indicators on the mental health of children and adolescents for application in public health surveillance—the current state in Germany and other OECD countries

The scientific and grey literature search initially extracted *N* = 7,477 indicators. Published articles and documents from Germany contributed the highest number of indicators (*N =* 2,427). It is important to note that our methodology, which had an additional focus on German grey literature, introduces a bias in these results. The other countries, ranked in decreasing order, with the highest number of published articles and documents from which indicators have been extracted were as follows: United States (*N =* 1818), Canada (*N =* 1,272), United Kingdom (*N =* 1,109), Spain (*N =* 1,058), Finland (*N =* 1,005), Australia (*N =* 959), Italy (*N =* 930), and Switzerland (*N =* 852).

Looking at publication timelines, publications on child and adolescent mental health indicators increased in the OECD region since 2008, as illustrated in Figure 1. In 2007, Bradshaw, Hoelscher, and Richardson emphasized the challenges in monitoring behavioral changes among children and adolescents, with limited exceptions (51). Similarly, Ben-Arieh’s study in the subsequent year drew attention to the initial emphasis on child wellbeing indicators, particularly focused on child survival (7). Additionally, in the same year, the European Commission launched the “Child Poverty and Well-being Report in the EU: Current Status and the Way Forward” (132). These influential contributions resulted in a significant increase in the number of documents published from around 2008, reflecting the growing recognition of the importance of monitoring and addressing the wellbeing of children and adolescents.

When comparing relative numbers and distributions by indicators of OECD in comparison to German indicators (as illustrated in Table 2), superordinate mental health topics such as “*psychological resources*,” “*social resources*,” “*social risks*,” “*positive mental health*,” and “*self-harm/suicidality*” were represented less frequently in the scope of the German included literature, in comparison to the international literature. In contrast, the superordinate topics “*mental health promotion/prevention*,” “*mental disorders*,” and especially “*supply and utilization*” (International 4.0%; Germany 15.2%) were found to be represented more frequently and mostly identified in grey literature documents when comparing the German included literature to the international literature. These findings apply to indicators identified for the population subgroup of children and adolescents and illustrate the proportional distribution of indicators in the respective settings. When looking into the total number of indicators and their distribution, solely focusing on the German setting, it can, however, be seen that, for example, indicators on “*mental disorders/psychopathology*” (*N* = 347) are more represented than, for example, “*mental health promotion/prevention*” *(N = 82)*, which is in line with the findings by Peitz and colleagues (20). This emphasizes the stronger focus on the care and rehabilitation of mental disorders compared to the prevention and promotion of mental health within Germany.

The included indicators in this scoping review exhibit a wide range of characteristics, varying from well-defined and currently utilized indicators for child and adolescent mental health surveillance to indicators exclusively suggested or discussed by government bodies, policymakers, scientists, and/or self-help groups. As a result, the operationalization level across the identified indicators is highly heterogeneous. Therefore, indicators were processed on the title level describing indicator concepts during this step of a scoping review (see Method section for explanation). While indicator concepts lacking sufficient information were excluded during the selection process, many of the included indicators still fall short of being readily applicable in routine monitoring, as defined by Peitz et al. (20). When considering the selection of an indicator or a comprehensive set of indicators for public health monitoring, numerous additional factors must be considered. In the absence of clear evidence, these factors may encompass aspects such as the background framework of the chosen indicator, including reliability (completeness, quality of monitoring, and data management, among others), independence of the indicator, validity (the extent to which the construct aligns with its intended outcome), predictive validity (based on longitudinal data), establishment of associations/correlations (concurrent validity), and the elucidation of causality, which remains uncertain. Furthermore, the strength of association, consideration of qualitative versus quantitative indicators (which may be easier to assess through proxy measures), and the measurement of complex items, such as relational aspects, may be important considerations in this process.

### 4.2 Current scientific gaps in reporting children and adolescents’ mental health reflected by unattended domains

Compared to adult indicators described by Peitz et al. (20), there are noticeable gaps in the level of indicators in children and adolescents’ mental health. While several corresponding indicators for adult mental health were found multiple times, i.e., in several documents in the search for children and adolescents, 22 indicator categories were only found once for children and adolescents. These indicators were, for example, “Anti-stigma Movement,” “Teachers/Other Responsible Adults Well-being,” “Availability/Access to weapons,” and “Prevalence of Impulse Control Disorders,” among others. The less frequent mention shows that the identified indicators may be very valuable but deserve further research. Conversely, 98 indicators were mentioned more frequently, 2–10 times in the literature, and 97 were represented by 10 notions. For example, though a lower level of aggregation was used within the indicators belonging to the superordinate topic “*supply and utilization*,”: many of the indicator categories were found 2–10 times, showing a more elaborated understanding and use of these indicators.

Most indicators in the included literature were not specifically assigned to individual age ranges. Instead, the literature often provided age ranges that indicated the age group in which a particular survey was conducted and not in which a specific indicator was applied. This resulted in several instances of “misclassification” when, for instance, adolescents were asked about being pre-term born; under the indicator “Perinatal and Antenatal Background,” the age range of 12–14 years of interviewees was recorded. On the other hand, if the data were derived from early childhood treatment records, the age range of 0–2 years was recorded for the same event, depending on the data type. As a result, the age ranges mentioned for the indicators in the literature, and subsequently in our Table 1, primarily reflect the age at the time of the interview rather than the age when the event took place.

Considering the limitation, the highest number of indicators was identified for the age group of 15–17 years (21.5%), followed by the age group of 12–14 years (21.3%), with a decreasing trend as the children’s age decreased. As such, the 0–2 years age group had the lowest proportion of indicators (7.0%) (see Figure 3). These results reflect the operational feasibility rather than addressing the monitoring needs of the respective population sub-group, particularly considering the vulnerability of younger children. Furthermore, it was observed that documents stated that certain indicators are applicable, e.g., for the 0–2 years age group; however, it is obvious that they are not specifically designed for younger children, or not applicable despite being stated as such. Examples of such indicators include “Bullying,” “Teen Pregnancy,” “Mental Health Locus of Control,” “Optimism,” “Self-Esteem,” “Self-Management,” “Leisure Time,” “Self-Harm,” or “Suicide Rate” (see Table 1). The presence of these indicators within these age groups raises concerns regarding the suitability of applying these concepts or diagnoses at such a young age. It is worth noting that there were significantly fewer indicators available for the vulnerable phases of early childhood and essential aspects of family dynamics and parenting during the initial years. This observation aligns with the United Nations Children’s Fund (UNICEF’s) 2009 report, which highlighted the disproportionate focus of many surveys on adolescents, such as the Program for International Student Assessment (PISA), the Health Behavior in School-aged Children (HBSC), and the International Civic and Citizenship Education Study (ICCS). The report further emphasizes the underrepresentation of young children in early childhood and primary school age in international data sources, as well as in research studies involving relevant constructs. Consequently, a comprehensive understanding of mental health (and wellbeing) among young children is limited, and, in many instances, age-appropriate measures are lacking (73). This hampers age-specific monitoring and, thus, evidence-based political efforts to support these vulnerable groups adequately.

Indicators such as “Breastfeeding,” “Cognitive Development and Function,” “Family Functionality,” and “Family Well-being,” as well as “Parenting Style and Skills,” could be considered age-relevant indicators. Our findings show that the indicator “Perinatal and Antenatal Background” including preterm birth, although having a significant impact on mental health development, is yet underrepresented. While 77 indicators were identified directly related to peri- and antenatal background, no specific category emerged addressing pregnant or young mothers’ mental health and wellbeing.

Finally, several findings emerge when examining the gaps between indicators identified in this scoping review and the WHO recommendations for mental health monitoring (*stated in italics*) (9):

*1) “Crucial information and indicators that are needed for the mental health system include the extent of the problem (the prevalence of mental disorders and identification of major risk factors and protective factors for mental health and well-being)[…]”* (9):

In terms of measuring the extent of the mental health problems, including the prevalence of mental disorders and identifying major risk and protective factors, we find that the data availability with the following identified superordinate topics ‘*mental disorder*’ (*N =* 1,168 indicators), ‘*social resources*’ (*N =* 1,120 indicators), and ‘*individual risks*’ (*N =* 907 indicators) was sufficient.

*2) “Coverage of policies and legislation, interventions and services (including the gap between the number of people who have a mental disorder and those who receive treatment and a range of appropriate services, such as social services)[…]”* (9).

Available data are limited when considering the coverage of policies and legislation (*N =* 166) and interventions (*N =* 429). ‘Services/supply and utilization’ accounted for *N =* 403 indicators. Specifically, for monitoring the gap between the number of people living with mental disorders and those who receive treatment and appropriate social services, we identified only (*N* = 6) indicators serving this purpose in the indicator category “needs, unmet needs and barriers in mental health care.”

*3) “Health outcome data (including suicide and premature mortality rates at the population level as well as individual- or group-level improvements related to clinical symptoms, levels of disability, overall functioning and quality of life)[…]”* (9):

Turning to health outcome data (such as represented by the indicators belonging to the superordinate topic “Self-Harm and Suicide”) as well as improvements in clinical symptoms identified by the indicators such as “Treatment Success,” “Inpatient Readmission by Mental Health Diagnosis,” “Quality of Mental Health Services,” “Levels of Disability” (no indicator identified), “General Mental Health status (i.e., overall Functioning), and “Health-related Quality of Life” (i.e., quality of life), “Life Satisfaction” as well as “Mental Well-being” revealed a mixed picture with significant gaps, especially in the area of the improvements of clinical symptoms and other health outcome data including the levels of disability. Further development of indicators may be required in this area.

*4)“Social and economic outcome data (including relative levels of educational achievement, housing, employment and income among persons with mental disorders)[…]”* (9).

Regarding social and economic outcome data, including indicators on “Childs/Adolescents Level of Education” (i.e., educational achievement), “Problematic Housing Conditions,” and “Family Socio-economic Situation (Employment Status, Household Income, etc.), data availability can be described as moderate.

In summary, in comparison to WHO recommendations, there are only limited indicators measuring the treatment gap and health outcome data, e.g., improvements in clinical symptoms, levels of disability, and gaps in policy coverage and social and economic outcome data.

## 5 Limitations and future research

While this scoping review was comprehensive and involved a substantial amount of data, several limitations should be acknowledged. The searches could not be standardized in the different databases; therefore, the search strategies are described in Supplementary material S2. The grey literature search of German institutions (in German) was an in-depth focus of the study in contrast to international institutions (in English) due to the aim to include the data to expand the current MHS for adults at RKI. Therefore, the number of German indicators found is disproportionally high compared to the other OECD countries, introducing a bias into the data. The international grey literature search was limited to English documents. Our direct email contact to institutions was written in English, which could explain why, of the 38 OECD countries contacted, only 23 replied. The grey literature search was conducted by the snowball system, starting by exploring key national institution web pages over links to documents and other institutions and direct contact with institutions. Therefore, the number of screened web pages and documents was not exhaustive. The keywords used for the different web pages were heterogeneous as they needed to be adapted to the different search options of the webpages. Keywords used for the searches were also a matter of debate as the translation from the English keywords used for the international search to key words used for the German search left gaps and uncertainties in the respective meaning and terminology usage. The use of Google and Google Scholar yielded slightly different results when repeating the searches, as the results took the search history into account. To compensate, we searched each search string in Google Scholar, and the first 160 hits were sorted by relevance, although the level of saturation was, on average, reached after 80–120 hits. Using Google for searches on websites of relevant institutions could not precisely be recorded; searches revealed different results and could not accurately be repeated. We accounted for this by stating the minimum of screened documents/websites. However, since the searches for both scientific articles and grey literature were extremely broad and thorough, the limitations should not affect the body of evidence retrieved and analyzed. A further quality element and critical appraisal was implemented by the exclusion of scientific publications of certain methodologies as described above under exclusion criteria. This approach aimed to ensure the inclusion of relevant and high-quality literature while excluding studies that did not meet the predefined criteria.

For further research and monitoring work, the following is suggested:

First, since indicators in the youngest age group (0–2) were less represented, upcoming studies and future monitoring efforts should focus on this age group to develop age-appropriate indicators. These indicators are crucial to monitor this vulnerable phase closely and detect trends and needs as soon as possible.

Second, indicators not found for children and adolescents, such as those on ‘*mental health literacy*’, should be further developed since the belonging concepts of, for example, knowledge about mental health as well as mental disorders and their stigmatization display important topics in this age groups as well and should be focused on public mental health efforts.

Third, the identified 71 new indicators may serve as a pivotal first step in expanding MHS efforts for children and youth, as this age group represents a vulnerable phase for the development of mental health problems. For example, a structured consensus process with stakeholders on the importance of these indicators from a public health perspective, as it has been done in Germany for adults (19), should be considered for children and adolescents as well. This consensus process should also include if the categorization orientated heavily towards adults is appropriate for children and adolescents.

Fourth, indicators that overlap for children and adolescents and overlap for children/adolescents and adults need to be further developed in terms of different measurements and operationalizations. As has been done in the present study, titles may need to be slightly adjusted (“Patient Satisfaction with Mental Health Care System” to “Patient/Parents or Family Satisfaction with Mental Health Care Services”).

Fifth, future research and monitoring work should focus in general on the indicators’ validity, for example, by investigating the different sides of validity and determining if certain indicators tap into similar aspects or have varying levels of usefulness. Therefore, different operational settings should be established and tested. Moreover, pragmatic issues and costs associated with sampling these indicators could also be explored to facilitate their implementation. Finally, investigating the sensitivity of indicators to changes in population mental health and subgroup characteristics should provide valuable insights.

In summary, while this scoping review contributes valuable findings on mental health monitoring indicators for children and adolescents, it is important to address the identified limitations and undertake further research to enhance the comprehensiveness, validity, and applicability of these indicators. By doing so, we can better monitor and understand the mental health needs of this age group and inform evidence-based interventions and policies.

## 6 Conclusion

In this scoping review, we made the following conclusions:

First, we identified 71 new indicators, reflecting an evolving landscape for children and adolescent MHS.
Second, articles and documents from Germany contributed—by methodology—the highest number of indicators, followed by the United States, Canada, the United Kingdom, Spain, Finland, Australia, Italy, and Switzerland.
Third, in the German document, indicators belonging to the mental health topics of “*psychological resources*” or “*social resources*” and “*positive mental health*” were identified less frequently compared to other OECD countries. Indicators related to “*self-harm and suicidality*” were also scarce, while indicators from the categories of “*mental health promotion/prevention*,” “*mental disorders/psychopathology*”, and “*supply and utilization*” were found more frequently by comparison to other OECD countries (which could also have been influenced by the search).
Fourth, within the German landscape, “*mental disorders/psychopathology*,” “*individual risks*,” and “*supply and utilization*” were the most prominent mental health topics, while “*participation*,” “*quality of care*,” “*costs*,” and measurement of “*preclinical symptoms*” were found less frequent. These findings reveal the need to emphasize these topics in further monitoring work in Germany.
Fifth, although we identified new indicators, many of them were only found once (i.e., represented by one record), including topics such as “Anti-stigma Movement” and “Inclusion of Family or Social Environment in Treatment,” or “Self-help Intervention Capacity.”
Sixth, indicators for children below 2 years of age were scarce, despite their vulnerability; in addition, the indicators we identified specific to this age group were often perceived by us as inappropriate for this age group.
Seventh, we did not find indicators specifically associated with the level of disability emphasizing the need to address mental health surveillance of children and adolescents with diverse requirements.
Eighth, in comparison to WHO recommendations, our findings highlighted that there are only limited indicators for improvements in clinical symptoms or treatment success. In line with WHO, we also emphasized the need to address gaps in policy coverage and social and economic outcome data.
Ninth, we stressed the importance of recognizing cultural specificity in surveillance indicators to ensure their validity and reliability across diverse populations. By addressing these gaps, we can enhance the comprehensiveness and effectiveness of mental health monitoring for children and adolescents.

In conclusion, our study provides valuable insights into the current state of mental health monitoring indicators for children and adolescents. However, further research and collaboration are needed to refine and expand the indicators, fill the identified gaps, and improve mental health surveillance for this vulnerable group.

## Data availability statement

The original contributions presented in the study are included in the article/Supplementary material, further inquiries can be directed to the corresponding author.

## Author contributions

AD: Conceptualization, Writing – review & editing, Data curation, Formal analysis, Investigation, Methodology, Resources, Visualization, Writing – original draft. SR-R: Conceptualization, Data curation, Formal analysis, Methodology, Resources, Visualization, Writing – original draft, Investigation, Writing – review & editing. TB: Investigation, Methodology, Writing – review & editing. OH: Investigation, Writing – review & editing, Methodology, Project administration, Supervision. AE: Writing – review & editing, Data curation, Formal analysis, Investigation. MO-P: Writing – review & editing, Data curation, Formal analysis, Investigation. CF: Data curation, Formal analysis, Investigation, Writing – review & editing. TM: Investigation, Methodology, Supervision, Writing – review & editing. LB: Formal analysis, Project administration, Supervision, Writing – review & editing. JT: Methodology, Supervision, Writing – review & editing. EM: Methodology, Writing – review & editing. DP: Conceptualization, Funding acquisition, Methodology, Project administration, Supervision, Validation, Writing – review & editing.

## Footnotes

^1^https://pubmed.ncbi.nlm.nih.gov/

^2^https://scholar.google.com/

^3^https://psych-info.de/

^4^https://www.cochranelibrary.com/

^5^Austria, Australia, Belgium, Canada, Chile, Colombia, Costa Rica, Czech Republic, Denmark, Estonia, Finland, France, Germany, Greece, Hungary, Iceland, Ireland, Israel, Italy, Japan, Korea, Latvia, Lithuania, Luxembourg, Mexico, the Netherland, New Zealand, Norway, Poland, Portugal, Slovakia, Slovenia, Spain, Sweden, Switzerland, Turkey, the United Kingdom, and the United States.

^6^https://www.rayyan.ai
