# Supplementary material for: Mental health indicators for children and adolescents in OECD countries: a scoping review
Source: Front Public Health. 2024 Feb 13;11:1303133. doi: 10.3389/fpubh.2023.1303133 (PMC10898649; doi:10.3389/fpubh.2023.1303133)
Supplement: Supplementary file 1 [file Data_Sheet_1.docx]

Supplement 1:

Mental Health Indicators for Children and Adolescents in OECD countries: A Scoping Review

**STUDY PROTOCOL**

# Background

Childhood and adolescence represent important and vulnerable periods in the life of every human being: They are characterized by developmental and emotional milestones, that individuals with good mental health reach, resulting in the acquisition of social as well as effective coping skills. This leads to a greater quality of life and adequate functioning in different domains of life^^[[1]](#endnote-2)^^.

However, these developmental periods are also particularly susceptible to disruptive factors. More than half of mental health problems start in childhood and adolescence, and tend to persist throughout adult life^^[[2]](#endnote-3)^^. Currently, almost 18% of the German population under 18 live with a mental disorder^^[[3]](#endnote-4)^^, while more than 20% of them receive no treatment^^[[4]](#endnote-5)^^. Moreover, the burden on individual lives (impairment in different life domains) as well as on society in general (direct and indirect costs) is high^^[[5]](#endnote-6)^^.

Considering these adverse effects, monitoring and promoting children and adolescents’ mental health possesses particular public health relevance. However, systematic monitoring as part of dedicated mental health surveillance (MHS) strategies for children and adolescents is scarce around the globe. MHS is intended to systematically collect, integrate and process data on the mental health of the population from different sources, as well as to analyze and interpret these data in order to report results on a regular base. The aim is to monitor the current state as well as trends in public mental health and to inform the evaluation of measures taken in mental health prevention, promotion and care. In other words, MHS output should be designed to serve as a reliable empirical foundation for evidence-based policy advice which enables political stakeholders to plan, initiate and assess necessary health political actions^^[[6]](#endnote-7)^^.

To date, one of the few examples of an MHS program addressing children and adolescents is in the USA^^[[7]](#endnote-8)^,^[[8]](#endnote-9)^^, which regularly synthesizes results from different independent federal surveillance systems, each addressing different health issues and varying populations^1,7^. Canada has also begun to include young people in its Positive Mental Health Surveillance^^[[9]](#endnote-10)^^.

In Germany, the data situation on the public mental health of children and adolescents is fragmented: There are several organizations providing data, but this data is often cross-sectional or not representative of the population^^[[10]](#endnote-11)^^. The “BEfragung zum seeLischen WohLbefinden und VerhAlten” (BELLA study) within the German Health Interview and Examination Survey for Children and Adolescents (KiGGS) managed by the Robert Koch Institute (RKI) is a cohort study offering longitudinal, nationally representative data on the mental health of this age group. However, different data sources need to be integrated and interpreted together to make informed statements on the mental health of children and adolescents and to support health political decision making in Germany.

The effectiveness of an MHS system depends on the careful selection of appropriate indicators which adequately capture population mental health. A broad overview of existing concepts and indicators currently used in the field of public mental health with regard to children and adolescents is required for establishing a solid basis for a future core indicator set for MHS in these age groups.

One of RKI´s MHS working group’s task is to monitor mental health in different population groups. Advancements have been done in the monitoring of mental health in Germany, especially with the establishment of a set core of indicators for the adult population based on a scoping review done in the first MHS funding phase. The same advancements should now be extended to monitor children and adolescents’ mental health.

This scoping review, following the PRISMA 2020 statement and its extension for scoping reviews (Page *et al.*, 2021)^^[[11]](#endnote-12)^^, aims to gain a holistic overview of current public mental health indicators for children and adolescents on the basis of the national (German) and international literature, with a focus on the OECD countries.

# Objective

The objective of this assignment is to critically review and summarize all available publications considering:

- Which indicators on the mental health of children and adolescents for application in public health surveillance can be identified on the basis of the current state of knowledge in Germany and other OECD countries?
- What are current scientific gaps and so-called “blind spots”?
- How can we address and further discuss the child and adolescent mental health gaps in current national and international monitoring work by taking an inclusive approach (which takes the perspectives of those affected into account)?

# Databases used for peer-reviewed scientific articles

- Pubmed-MEDLINE: https://pubmed.ncbi.nlm.nih.gov/
- Google Scholar: https://scholar.google.com/
- PsychInfo
- Cochrane database for systematic reviews

# Grey literature:

- World Bank: https://datacatalog.worldbank.org/
- WHO: European Health for all Databases (WHO Regional Office for Europe) - https://gateway.euro.who.int/en/how-to/hfa/how-to-find-an-indicator-in-the-european-health-for-all-family-of-databases/ and https://apps.who.int/iris/
- OECD: https://www.oecd-ilibrary.org/search/advancedsearch
- Websites of international organizations [European Union (EU), WHO, and Organization for Economic Co-operation and Development (OECD)] for supranational documents
- Public health institutes of the 38 OECD member countries via member list of the “International Association of National Public Health Institutes” (www.ianphi.org) or by google search (see below).
- Search in German on the websites of selected national stakeholders of mental health care, such as professional associations, service providers and federal agencies (KBV/Kassenärztliche Bundesvereinigung); DGPPN/Deutsche Gesellschaft für Psychotherapie, Psychosomatik und Bundespsychotherapeutenkammer, Psychisch Kranke e.V.; GKV; Spitzenverband/Spitzenverband Bund der Krankenkassen, AOLG/Arbeitsgemeinschaft der Obersten Landesgesundheitsbehörden; Fachgesellschaften, Selbsthilfegruppen, Betroffenenvertretungen
- Include German population-based surveys/cohorts (KIGGS, KIDA, NaKo, Twinlife, etc.)
- Keyword search in German using Google for indicators of “mental health care” and “mental health care research”, “mental health care prevention” and “mental health care promotion” in Germany.
- Bibliographies of all included documents, which were found in the procedure mentioned above

# Search strategy

The search will be limited to literature from 01.01.2000 onwards. The search will be performed in English however aim to not exclude any language retrieved. An in-depth search will be performed in German language specifically for German Institutions.

The specific searches will be adapted to the respective database.

The search will be derived from search terms and variations/synonyms covering the following categories:

1. “Indicators/monitoring/surveillance” at population level
2. “mental/psychological”
3. ” health/disorders”
4. “children and adolescents”,
5. “38 OECD countries”.

The variations of terms of the same category will be combined with the Boolean operator OR, the categories in brackets will then be combined with the Boolean operator AND.

## For PubMed-MEDLINE:

A broad search strategy will be applied around the above stated categories. The search will be optimized using the “advanced search tool” of PubMed and by using MeSH terms, free-text terms or limitations to title and abstract as perceived appropriate.

In PubMed, articles are indexed with the MeSH (medical sub-heading) terms which enable to distinguish between relevant articles and articles where the keywords appear somewhere but in an irrelevant context. Hence, using MeSH terms helps to pre-filter relevant articles in complex search strategies. The disadvantage of MeSH terms is, that the most recent articles might not have been indexed yet. Therefore, we decided to sub-divide the search string into two blocks, one using only MeSH-terms before 2022, and one using a combination of MeSH-terms and automated title-abstract in 2022.

**I) Search string for articles published before 2022:**

*(Indicator*[tiab] OR syndromic[tiab] OR "Behavioral Risk Factor Surveillance System"[MeSH] OR “Population surveillance”[MeSH Terms] OR “Epidemiological Monitoring” [MeSH] OR "Information Systems"[MeSH] OR "Sentinel Surveillance"[Mesh] OR "Public Health Surveillance"[MeSH])*

***AND***

*("mental disorders"[MeSH Terms] OR "mental health"[MeSH Terms] OR well-being[tiab] OR "Quality of Life"[Mesh] OR "Resilience, Psychological"[Mesh] OR "Codependency, Psychological"[Mesh] OR "Drug-Seeking Behavior"[Mesh] OR "Illness Behavior"[Mesh] OR "Self-Injurious Behavior"[Mesh] OR "Psychology"[Mesh] OR “Psychiatry"[Mesh] OR*

*(*

*("Primary Prevention"[Mesh] OR “secondary prevention”[MeSH] OR “tertiary prevention”[MeSH] OR "Health Promotion"[Mesh])*

***AND***

*("mental health"[MeSH Terms] OR “mental disorders”[MeSH])*

*)*

*)*

***AND***

*(“child”[MeSH] OR “infant”[MeSH] OR “adolescent”[MeSH] OR "Puberty"[Mesh])*

***AND***

*(Austria[MeSH] OR Australia[MeSH] OR Belgium[MeSH] OR Canada[MeSH] OR Chile[MeSH] OR Colombia[MeSH] OR “Costa Rica”[MeSH] OR “Czech Republic”[MeSH] OR Denmark[MeSH] OR Estonia[MeSH] OR Finland[MeSH] OR France[MeSH] OR Germany[MeSH] OR Greece[MeSH] OR Hungary[MeSH] OR Iceland[MeSH] OR Ireland[MeSH] OR Israel[MeSH] OR Italy[MeSH] OR Japan[MeSH] OR Korea[MeSH] OR Latvia[MeSH] OR Lithuania[MeSH] OR Luxembourg[MeSH] OR Mexico[MeSH] OR Netherland[MeSH] OR New Zealand[MeSH] OR Norway[MeSH] OR Poland[MeSH] OR Portugal[MeSH] OR Slovakia[MeSH] OR Slovenia[MeSH] OR Spain[MeSH] OR Sweden[MeSH] OR Switzerland[MeSH] OR Turkey[MeSH] OR United Kingdom[MeSH] OR United States[MeSH] OR "Organisation for Economic Co-Operation and Development"[Mesh])*

***AND***

*("2000/01/01"[Date - Publication] : "3000"[Date - Publication])*

**II) Search string for the year 2022:**

*(Surveillance[tiab] OR Monitoring[tiab] OR monitor[tiab] OR sentinel[tiab] OR Information system*[tiab] OR Indicator*[tiab] OR syndromic[tiab] OR "Behavioral Risk Factor Surveillance System"[MeSH] OR “Population surveillance”[MeSH Terms] OR “Epidemiological Monitoring” [MeSH] OR "Information Systems"[MeSH] OR "Sentinel Surveillance"[Mesh] OR "Public Health Surveillance"[MeSH])*

***AND***

*(*

*(Mental*[tiab] OR psychologic*[tiab] OR psychiatric*[tiab] OR behavio*[tiab])*

***AND***

*(Health[tiab] OR disorder*[tiab] OR problem*[tiab] OR ill[tiab] OR illness[tiab] OR sick*[tiab] OR disease*[tiab] OR constitution*[tiab] OR condition*[tiab] OR affected[tiab] OR suffer*[tiab] OR distress*[tiab] OR difficult*[tiab] OR disturbance*[tiab] OR strength*[tiab] OR competence*[tiab])*

***OR*** *("mental disorders"[MeSH Terms] OR "mental health"[MeSH Terms] OR “well-being”[tiab] OR "Quality of Life"[MeSH] OR "Resilience, Psychological"[MeSH] OR "Codependency, Psychological"[MeSH] OR "Drug-Seeking Behavior"[MeSH] OR "Illness Behavior"[MeSH] OR “Self-Injurious Behavior"[Mesh] OR "Psychology"[MeSH] OR “Psychiatry"[Mesh] OR*

*(*

*("Primary Prevention"[MeSH] OR “secondary prevention”[MeSH] OR “tertiary prevention”[MeSH] OR "Health Promotion"[Mesh])*

***AND***

*("mental health"[MeSH] OR “mental disorders”[MeSH]))*

*)*

*)*

***AND***

*(Child[tiab] OR childhood[tiab] OR children[tiab] OR adolescen*[tiab] OR youth[tiab] OR teen*[tiab] OR puberty[tiab] OR pupil*[tiab] OR infant*[tiab] OR “child”[MeSH] OR “infant”[MeSH] OR “adolescent”[MeSH] OR "Puberty"[Mesh])*

***AND***

*(Austria OR Australia OR Belgium OR Canada OR Chile OR Colombia OR “Costa Rica” OR “Czech Republic” OR Denmark OR Estonia OR Finland OR France OR Germany OR Greece OR Hungary OR Iceland OR Ireland OR Israel OR Italy OR Japan OR Korea OR Latvia OR Lithuania OR Luxembourg OR Mexico OR Netherland OR New Zealand OR Norway OR Poland OR Portugal OR Slovakia OR Slovenia OR Spain OR Sweden OR Switzerland OR Turkey OR United Kingdom OR United States)*

***AND***

*("2022/01/01"[Date - Publication]: "3000"[Date - Publication])*

## For Google scholar, PsychInfo and Cochrane

The search will be performed applying the main keywords from the categories of the search string above. In case of limitations due the search function the search will be divided into different substrings. The hits will be sorted by relevance. Depending on the complexity of the substrings and the number of gained relevant hits, a certain number of hits will be screened until a level of saturation is reached, for example screening the first 100 hits per search and add another 50 if in hit 70-100 a relevant document was detected. In order to reduce search terms, the search will be performed globally but selection will be limited to publications of OECD countries, or in case of supranational documents or international literature, to subsets used or considered for OECD countries.

## For grey literature

A search beyond the member list of the “International Association of National Public Health Institutes” (www.ianphi.org) will be performed via Google for relevant (public) mental health institutions in the OECD countries. Grey literature such as reports, health monitoring systems, and other documents will be screened with a focus on discussed, considered, or applied, country-specific mental health indicators.

- Selected national stakeholders of mental health care (e.g., professional associations, service providers, federal agencies): “Mental Disorders”, “Mental health promotion”, “Mental health prevention”.
- International organizations (EU, WHO, OECD): “Mental Health Surveillance;” “Mental Health Indicators;” “Mental Health Monitoring”, “Mental health promotion”, “Mental health prevention”.
- For each OECD country on www.ianphi.org: “Mental Health Surveillance;” “Mental Health Indicators;” “Mental Health Monitoring”, “Mental health promotion”, “Mental health prevention”.
- For each OECD listed public health institute not registered on The International Association of National Public Health Institutes (www.ianphi.org) “[Country] Mental Health Surveillance;” “[Country] Mental Health Indicators;” “[Country] Mental Health Monitoring” OR “Mental health promotion”, OR “Mental health prevention”

German search via google for German institutions:

- For relevant indicators on national public mental health care and mental health care research for example: “Versorgungsforschung”; “psych∗ Versorgung Bericht”; “psych∗ Versorgungssituation”; “psych∗ Versorgungsepidemiologie”. (translation: “∗health care research”; “psych∗ health care report”; “psych∗ health care situation”; “psych∗ epidemiology of health care”)

The institutions will first be researched on the internet and then contacted by email in a second step, if necessary. We will follow up on these emails, but we will stop following up and accepting responses on 1 November in order to meet the December deadline for the report.

# Eligibility criteria

All types of published information (such as websites, reports, scientific as well as official working and consultation papers, policy briefs, health strategy documents) will be screened with a particular focus on indicators used to monitor mental health of children and adolescents at a general population level in OECD member states.

# Inclusion criteria

1. focus on public mental health of
2. children and/or adolescents
3. focus on public health monitoring (in contrast to e.g., clinical research or case studies);
4. current data (date of publication after 01.01.2000).
5. OECD countries or supranational

# Exclusion criteria

1. Any data **if not** concerned with the general population or age specific sub-groups of the population ( if the document has no public health focus).
2. Data with a focus on somatic public health (instead of mental public health)
3. Case series, case studies, case reports, reviews without methodology, letters to the editors, editorials, comments

The exclusion criteria might be adjusted during the search process, depending on the findings.

# Selection of sources of evidence

The reference manager Endnote will be used for collecting the search results and to remove duplicates. Then, the remaining results will be exported into Rayyan, a software used for systematic reviews, for title and abstract screening. The imported documents will be screened by title and/or title and abstract and the preliminary included documents further assessed by full application of in- and exclusion criteria by two independent reviewers. Disagreement will be solved by agreement after discussion between both or in case of ongoing disagreement by consulting with the pool of experts. The selection process will be illustrated in a PRISMA flowchart.

# Data collection process and data analysis

Data will be extracted, using a pre-piloted data extraction form which takes the MHS framework into account to further identify a) “blind spots”, b) indicators which are not fitting into the MHS framework developed for adult mental health and therefor might need additional categories. The final topics/categories will be developed after a full-text assessment of the documents. Preliminary categories would be

1. authorship, publication year, complete citation
2. location, country, application setting (supranational, national, reginal local, other subgroups?)
3. Type of document (scientific or report -like, if scientific: methodology, study design of paper)
4. indicator name, indicator definition (if available measurement and operationalization)
5. indicator type, whether the indicator was already contained in a surveillance resp. monitoring system (in use or reported only); if yes, which one?
6. data (survey [representative cross sectional, longitudinal, cohort, other], routine, etc.]
7. Surveillance purpose specified (general monitoring, sentinel, detect early changes in trend, others)
8. Level of evidence behind an indicator. This might be if the indicator was evaluated, or if available the experience with an indicator as e.g. usefulness, applicability etc.
9. Age range in which the indicator was applied/considered

# Indicator selection process

After data extraction the indicators will be categorized in line with the MHS framework and excluded when:

(1) they are duplicates or monitoring a similar content

(2) they are not relevant or feasible for continued and population-based monitoring

(3) they are not supportive for mental health

Evidence tables will be created and further developed into result tables. Documents might be then grouped considering specific aspects e.g. quality of the document (level of evidence), the purpose of indicator, or level of implementation of indicators, the MHS framework will be a guiding instrument. Child and adolescent mental health gaps in current national and international monitoring work will be addressed and further discussed by taking an inclusive approach. Further research recommendations will be provided.

# Evaluation: Quality assessment

The selected literature will not be graded due to the scoping-review approach, however, a quality element could potentially be implemented by the application of additional inclusion or exclusion criteria based on a certain methodological quality or standard in order to “[systematically examine (the) research evidence in order to assess its validity, results, and relevance](https://www.prisma-statement.org/documents/PRISMA-ScR-Fillable-Checklist_11Sept2019.pdf)” as described in the PRISMA-ScR Checklist for scoping reviews.

# References

1. Perou R, Bitsko RH, Blumberg SJ, Pastor P, Ghandour RM, Gfroerer JC, et al. Mental health surveillance among children--United States, 2005-2011. MMWR Suppl. 2013;62(2):1-3 [↑](#endnote-ref-2)
2. Kessler RC, Berglund P, Demler O, Jin R, Merikangas KR, Walters EE. Lifetime prevalence and age-of-onset distributions of DSM-IV disorders in the National Comorbidity Survey Replication. Arch Gen Psychiatry. 2005;62(6):593-602 [↑](#endnote-ref-3)
3. Barkmann C, Schulte-Markwort M. Prevalence of emotional and behavioural disorders in German children and adolescents: a meta-analysis. J Epidemiol Community Health. 2012;66(3):194-203. [↑](#endnote-ref-4)
4. Otto C, Reiss F, Voss C, Wustner A, Meyrose AK, Holling H, et al. Mental health and well-being from childhood to adulthood: design, methods and results of the 11-year follow-up of the BELLA study. Eur Child Adolesc Psychiatry. 2021;30(10):1559-77 [↑](#endnote-ref-5)
5. Olesen J, Gustavsson A, Svensson M, Wittchen HU, Jonsson B, group Cs, et al. The economic cost of brain disorders in Europe. Eur J Neurol. 2012;19(1):155-62. [↑](#endnote-ref-6)
6. Choi BC. The past, present, and future of public health surveillance. Scientifica (Cairo). 2012;2012:875253. [↑](#endnote-ref-7)
7. Bitsko RH, Claussen AH, Lichstein J, Black LI, Jones SE, Danielson ML, et al. Mental Health Surveillance Among Children - United States, 2013-2019. MMWR Suppl. 2022;71(2):1-42. [↑](#endnote-ref-8)
8. [↑](#endnote-ref-9)
9. Public Health Agency of Canada CfSaAR. Positive Mental Health Indicator Framework Quick Statistics, youth (12 to 17 years of age), Canada, 2022 Edition. 2022. [↑](#endnote-ref-10)
10. Thom J, Mauz E, Peitz D, Kersjes C, Aichberger M, Baumeister H, et al. Establishing a Mental

    Health Surveillance in Germany: Development of a framework concept and indicator set. J Health Monit. 2021;6(4):34-63. [↑](#endnote-ref-11)
11. Page MJ, McKenzie JE, Bossuyt PM, Boutron I, Hoffmann TC, Mulrow CD, Shamseer L, Tetzlaff JM, Akl EA, Brennan SE, Chou R, Glanville J, Grimshaw JM, Hróbjartsson A, Lalu MM, Li T, Loder EW, Mayo-Wilson E, McDonald S, McGuinness LA, Stewart LA, Thomas J, Tricco AC, Welch VA, Whiting P, Moher D. The PRISMA 2020 statement: an updated guideline for reporting systematic reviews. BMJ. 2021 Mar 29;372:n71. doi: 10.1136/bmj.n71. PMID: 33782057; PMCID: PMC8005924 [↑](#endnote-ref-12)
