# Supplementary material for: Mental health indicators for children and adolescents in OECD countries: a scoping review
Source: Front Public Health. 2024 Feb 13;11:1303133. doi: 10.3389/fpubh.2023.1303133 (PMC10898649; doi:10.3389/fpubh.2023.1303133)
Supplement: Supplementary file 2 [file Data_Sheet_2.docx]

Supplement 2:

Mental Health Indicators for Children and Adolescents in OECD countries: A Scoping Review

**SEARCH STRATEGY**

# Search Strategy

## PubMed Search string for articles published before 2022:

*(Indicator*[tiab] OR syndromic[tiab] OR "Behavioral Risk Factor Surveillance System"[MeSH] OR “Population surveillance”[MeSH Terms] OR “Epidemiological Monitoring” [MeSH] OR "Information Systems"[MeSH] OR "Sentinel Surveillance"[Mesh] OR "Public Health Surveillance"[MeSH])*

***AND***

*("mental disorders"[MeSH Terms] OR "mental health"[MeSH Terms] OR well-being[tiab] OR "Quality of Life"[Mesh] OR "Resilience, Psychological"[Mesh] OR "Codependency, Psychological"[Mesh] OR "Drug-Seeking Behavior"[Mesh] OR "Illness Behavior"[Mesh] OR "Self-Injurious Behavior"[Mesh] OR "Psychology"[Mesh] OR “Psychiatry"[Mesh] OR*

*(*

*("Primary Prevention"[Mesh] OR “secondary prevention”[MeSH] OR “tertiary prevention”[MeSH] OR "Health Promotion"[Mesh])*

***AND***

*("mental health"[MeSH Terms] OR “mental disorders”[MeSH])*

*)*

*)*

***AND***

*(“child”[MeSH] OR “infant”[MeSH] OR “adolescent”[MeSH] OR "Puberty"[Mesh])*

***AND***

*(Austria[MeSH] OR Australia[MeSH] OR Belgium[MeSH] OR Canada[MeSH] OR Chile[MeSH] OR Colombia[MeSH] OR “Costa Rica”[MeSH] OR “Czech Republic”[MeSH] OR Denmark[MeSH] OR Estonia[MeSH] OR Finland[MeSH] OR France[MeSH] OR Germany[MeSH] OR Greece[MeSH] OR Hungary[MeSH] OR Iceland[MeSH] OR Ireland[MeSH] OR Israel[MeSH] OR Italy[MeSH] OR Japan[MeSH] OR Korea[MeSH] OR Latvia[MeSH] OR Lithuania[MeSH] OR Luxembourg[MeSH] OR Mexico[MeSH] OR Netherland[MeSH] OR New Zealand[MeSH] OR Norway[MeSH] OR Poland[MeSH] OR Portugal[MeSH] OR Slovakia[MeSH] OR Slovenia[MeSH] OR Spain[MeSH] OR Sweden[MeSH] OR Switzerland[MeSH] OR Turkey[MeSH] OR United Kingdom[MeSH] OR United States[MeSH] OR "Organisation for Economic Co-Operation and Development"[Mesh])*

***AND***

*("2000/01/01"[Date - Publication] : "3000"[Date - Publication])*

## PubMed Search string for the year 2022:

*(Surveillance[tiab] OR Monitoring[tiab] OR monitor[tiab] OR sentinel[tiab] OR Information system*[tiab] OR Indicator*[tiab] OR syndromic[tiab] OR "Behavioral Risk Factor Surveillance System"[MeSH] OR “Population surveillance”[MeSH Terms] OR “Epidemiological Monitoring” [MeSH] OR "Information Systems"[MeSH] OR "Sentinel Surveillance"[Mesh] OR "Public Health Surveillance"[MeSH])*

***AND***

*(*

*(Mental*[tiab] OR psychologic*[tiab] OR psychiatric*[tiab] OR behavio*[tiab])*

***AND***

*(Health[tiab] OR disorder*[tiab] OR problem*[tiab] OR ill[tiab] OR illness[tiab] OR sick*[tiab] OR disease*[tiab] OR constitution*[tiab] OR condition*[tiab] OR affected[tiab] OR suffer*[tiab] OR distress*[tiab] OR difficult*[tiab] OR disturbance*[tiab] OR strength*[tiab] OR competence*[tiab])*

***OR*** *("mental disorders"[MeSH Terms] OR "mental health"[MeSH Terms] OR “well-being”[tiab] OR "Quality of Life"[MeSH] OR "Resilience, Psychological"[MeSH] OR "Codependency, Psychological"[MeSH] OR "Drug-Seeking Behavior"[MeSH] OR "Illness Behavior"[MeSH] OR “Self-Injurious Behavior"[Mesh] OR "Psychology"[MeSH] OR “Psychiatry"[Mesh] OR*

*(*

*("Primary Prevention"[MeSH] OR “secondary prevention”[MeSH] OR “tertiary prevention”[MeSH] OR "Health Promotion"[Mesh])*

***AND***

*("mental health"[MeSH] OR “mental disorders”[MeSH]))*

*)*

*)*

***AND***

*(Child[tiab] OR childhood[tiab] OR children[tiab] OR adolescen*[tiab] OR youth[tiab] OR teen*[tiab] OR puberty[tiab] OR pupil*[tiab] OR infant*[tiab] OR “child”[MeSH] OR “infant”[MeSH] OR “adolescent”[MeSH] OR "Puberty"[Mesh])*

***AND***

*(Austria OR Australia OR Belgium OR Canada OR Chile OR Colombia OR “Costa Rica” OR “Czech Republic” OR Denmark OR Estonia OR Finland OR France OR Germany OR Greece OR Hungary OR Iceland OR Ireland OR Israel OR Italy OR Japan OR Korea OR Latvia OR Lithuania OR Luxembourg OR Mexico OR Netherland OR New Zealand OR Norway OR Poland OR Portugal OR Slovakia OR Slovenia OR Spain OR Sweden OR Switzerland OR Turkey OR United Kingdom OR United States)*

***AND***

*("2022/01/01"[Date - Publication]: "3000"[Date - Publication])*

## PsycINFO search string

Before 2022:

(*Indicator*.ti,ab. OR syndromic.ti,ab. OR MM "Disease Surveillance" OR MM "Monitoring" OR MM "Polysomnography" OR MM "Self-Monitoring" OR MM "Vigilance" OR MM "Information Systems")****AND*** *(MM "Mental Disorders" OR MM "Affective Disorders" OR MM "Anxiety Disorders" OR MM "Autism Spectrum Disorders" OR MM "Bipolar Disorder" OR MM "Borderline States" OR MM "Chronic Mental Illness" OR MM "Dissociative Disorders" OR MM "Eating Disorders" OR MM "Gender Dysphoria" OR MM "Mental Disorders due to General Medical Conditions" OR MM "Neurocognitive Disorders" OR MM "Neurodevelopmental Disorders" OR MM "Neurosis" OR MM "Paraphilias" OR MM "Personality Disorders" OR MM "Psychosis" OR MM "Serious Mental Illness" OR MM "Sleep Wake Disorders" OR MM "Somatoform Disorders" OR MM "Stress and Trauma Related Disorders" OR MM "Substance Related and Addictive Disorders" OR MM "Thought Disturbances" OR MM "Mental Health" OR MM "Mental Health Disparities" OR MM "Well Being" OR MM "Quality of Life" OR MM "Health Related Quality of Life" OR MM "Resilience (Psychological)"  OR MM "Codependency" OR MM "Drug Abuse" OR MM "Inhalant Abuse" OR MM "Polydrug Abuse" OR MM "Illness Behavior" OR MM "Somatization" OR MM "Self-Injurious Behavior" OR MM "Self-Inflicted Wounds" OR MM "Self-Mutilation" OR MM "Self-Poisoning" OR MM "Self-Destructive Behavior" OR MM "Self-Injurious Behavior" OR MM "Suicidal Behavior"  OR MM "Psychology" OR MM "Psychiatry" OR MM "Adolescent Psychiatry" OR MM "Preventive Mental Health Services" OR MM "Mental Health Programs" OR MM "Crisis Intervention Services" OR MM "Suicide Prevention" OR MM "Violence Prevention" OR MM "Mental Health Services" OR MM "Psychological First Aid" OR MM "School Based Mental Health Services" OR DE "School Based Mental Health Services" OR DE "Public Mental Health" OR MM "Crime Prevention" OR MM "Substance Use Prevention OR*

*(*

*(MM "Prevention" OR MM "Preventive Health Behavior" OR MM "Preventive Health Services" OR MM "Relapse Prevention")* ***AND*** *(MM "Mental Disorders" OR MM "Mental Health" OR MM "Affective Disorders" OR MM "Anxiety Disorders" OR MM "Autism Spectrum Disorders" OR MM "Bipolar Disorder" OR MM "Borderline States" OR MM "Chronic Mental Illness" OR MM "Dissociative Disorders" OR MM "Eating Disorders" OR MM "Gender Dysphoria" OR MM "Mental Disorders due to General Medical Conditions" OR MM "Neurocognitive Disorders" OR MM "Neurodevelopmental Disorders" OR MM "Neurosis" OR MM "Paraphilias" OR MM "Personality Disorders" OR MM "Psychosis" OR MM "Serious Mental Illness" OR MM "Sleep Wake Disorders" OR MM "Somatoform Disorders" OR MM "Stress and Trauma Related Disorders" OR MM "Substance Related and Addictive Disorders" OR MM "Thought Disturbances")
 )
)****AND*** *(child OR infant OR adolescent OR Puberty)****AND*** *(Austria OR Australia OR Belgium OR Canada OR Chile OR Colombia OR "Costa Rica" OR "Czech Republic" OR Denmark OR Estonia OR Finland OR France OR Germany OR Greece OR Hungary OR Iceland OR Ireland OR Israel OR Italy OR Japan OR Korea OR Latvia OR Lithuania OR Luxembourg OR Mexico OR Netherland OR "New Zealand" OR Norway OR Poland OR Portugal OR Slovakia OR Slovenia OR Spain OR Sweden OR Switzerland OR Turkey OR "United Kingdom" OR "United States" OR "Organisation for Economic Co-Operation and Development")*

2022:

*(Indicator*.ti,ab. OR syndromic.ti,ab. OR MM "Disease Surveillance" OR MM "Monitoring" OR MM "Polysomnography" OR MM "Self-Monitoring" OR MM "Vigilance" OR MM "Information Systems" OR Behavioral Risk Factor Surveillance System OR Epidemiological Monitoring OR Information System* OR Sentinel OR Surveillance)****AND*** *(MM "Mental Disorders" OR MM "Affective Disorders" OR MM "Anxiety Disorders" OR MM "Autism Spectrum Disorders" OR MM "Bipolar Disorder" OR MM "Borderline States" OR MM "Chronic Mental Illness" OR MM "Dissociative Disorders" OR MM "Eating Disorders" OR MM "Gender Dysphoria" OR MM "Mental Disorders due to General Medical Conditions" OR MM "Neurocognitive Disorders" OR MM "Neurodevelopmental Disorders" OR MM "Neurosis" OR MM "Paraphilias" OR MM "Personality Disorders" OR MM "Psychosis" OR MM "Serious Mental Illness" OR MM "Sleep Wake Disorders" OR MM "Somatoform Disorders" OR MM "Stress and Trauma Related Disorders" OR MM "Substance Related and Addictive Disorders" OR MM "Thought Disturbances" OR MM "Mental Health" OR MM "Mental Health Disparities" OR MM "Well Being" OR MM "Quality of Life" OR MM "Health Related Quality of Life" OR MM "Resilience (Psychological)"  OR MM "Codependency" OR MM "Drug Abuse" OR MM "Inhalant Abuse" OR MM "Polydrug Abuse" OR MM "Illness Behavior" OR MM "Somatization" OR MM "Self-Injurious Behavior" OR MM "Self-Inflicted Wounds" OR MM "Self-Mutilation" OR MM "Self-Poisoning" OR MM "Self-Destructive Behavior" OR MM "Self-Injurious Behavior" OR MM "Suicidal Behavior"  OR MM "Psychology" OR MM "Psychiatry" OR MM "Adolescent Psychiatry" OR MM "Preventive Mental Health Services" OR MM "Mental Health Programs" OR MM "Crisis Intervention Services" OR MM "Suicide Prevention" OR MM "Violence Prevention" OR MM "Mental Health Services" OR MM "Psychological First Aid" OR MM "School Based Mental Health Services" OR DE "School Based Mental Health Services" OR DE "Public Mental Health" OR MM "Crime Prevention" OR MM "Substance Use Prevention OR mental disorders OR mental health/ OR well-being.ti,ab. OR “Quality of Life" OR Resilience/ OR Codependency OR Drug-Seeking Behavior OR Illness Behavior OR exp Self-Injurious Behavior OR exp Psychology OR exp Psychiatry OR*

*(*

*(MM "Prevention" OR MM "Preventive Health Behavior" OR MM "Preventive Health Services" OR MM "Relapse Prevention" OR Prevention OR Health Promotion)* ***AND*** *(MM "Mental Disorders" OR MM "Mental Health" OR MM "Affective Disorders" OR MM "Anxiety Disorders" OR MM "Autism Spectrum Disorders" OR MM "Bipolar Disorder" OR MM "Borderline States" OR MM "Chronic Mental Illness" OR MM "Dissociative Disorders" OR MM "Eating Disorders" OR MM "Gender Dysphoria" OR MM "Mental Disorders due to General Medical Conditions" OR MM "Neurocognitive Disorders" OR MM "Neurodevelopmental Disorders" OR MM "Neurosis" OR MM "Paraphilias" OR MM "Personality Disorders" OR MM "Psychosis" OR MM "Serious Mental Illness" OR MM "Sleep Wake Disorders" OR MM "Somatoform Disorders" OR MM "Stress and Trauma Related Disorders" OR MM "Substance Related and Addictive Disorders" OR MM "Thought Disturbances" OR mental health OR mental disorders)
 )
)****AND*** *(child OR infant OR adolescent OR Puberty)****AND*** *(Austria OR Australia OR Belgium OR Canada OR Chile OR Colombia OR "Costa Rica" OR "Czech Republic" OR Denmark OR Estonia OR Finland OR France OR Germany OR Greece OR Hungary OR Iceland OR Ireland OR Israel OR Italy OR Japan OR Korea OR Latvia OR Lithuania OR Luxembourg OR Mexico OR Netherland OR "New Zealand" OR Norway OR Poland OR Portugal OR Slovakia OR Slovenia OR Spain OR Sweden OR Switzerland OR Turkey OR "United Kingdom" OR "United States" OR "Organisation for Economic Co-Operation and Development")*

## COCHRANE Search String

*((Indicator* OR syndromic OR “Behavioral Risk Factor Surveillance System” OR Surveillance OR “Epidemiological Monitoring” OR "Information System*" OR Sentinel)*

***AND***

*(mental OR well-being OR "Quality of Life" OR Resilience OR Codependency OR "Drug-Seeking Behavior" OR "Illness Behavior" OR "Self-Injurious Behavior" OR Psychology OR Psychiatry OR ((Prevention OR "Health Promotion") AND (mental)))*

***AND***

*(child OR infant OR adolescent* OR Puberty)*

***AND***

*(Austria OR Australia OR Belgium OR Canada OR Chile OR Colombia OR “Costa Rica” OR “Czech Republic” OR Denmark OR Estonia OR Finland OR France OR Germany OR Greece OR Hungary OR Iceland OR Ireland OR Israel OR Italy OR Japan OR Korea OR Latvia OR Lithuania OR Luxembourg OR Mexico OR Netherland OR New Zealand OR Norway OR Poland OR Portugal OR Slovakia OR Slovenia OR Spain OR Sweden OR Switzerland OR Turkey OR United Kingdom OR United States OR "Organisation for Economic Co-Operation and Development" OR OECD)):ti,ab,kw*

## Google Scholar Search Strings

1. *(Indicator* OR Monitoring OR Surveillance)* ***AND*** *(“mental health” OR “mental disorder*” OR well-being OR Resilience OR behavior* OR prevention OR promotion)* ***AND*** *(child OR infant OR adolescent OR Puberty)* ***AND*** *(Austria OR Australia OR Belgium)*
2. *(Indicator* OR Monitoring OR Surveillance)* ***AND*** *(“mental health” OR “mental disorder*” OR well-being OR Resilience OR behavior* OR prevention OR promotion)* ***AND*** *(child OR infant OR adolescent OR Puberty)* ***AND*** *(Canada OR Chile OR Colombia)*
3. *(Indicator* OR Monitoring OR Surveillance)* ***AND*** *(“mental health” OR “mental disorder*” OR well-being OR Resilience OR behavior* OR prevention OR promotion)* ***AND*** *(child OR infant OR adolescent OR Puberty)* ***AND*** *(“Costa Rica” OR “Czech Republic”)*
4. *(Indicator* OR Monitoring OR Surveillance)* ***AND*** *(“mental health” OR “mental disorder*” OR well-being OR Resilience OR behavior* OR prevention OR promotion)* ***AND*** *(child OR infant OR adolescent OR Puberty)* ***AND*** *(Denmark OR Estonia OR Finland)*
5. *(Indicator* OR Monitoring OR Surveillance)* ***AND*** *(“mental health” OR “mental disorder*” OR well-being OR Resilience OR behavior* OR prevention OR promotion)* ***AND*** *(child OR infant OR adolescent OR Puberty)* ***AND*** *(France OR Germany OR Greece OR Hungary)*
6. *(Indicator* OR Monitoring OR Surveillance)* ***AND*** *(“mental health” OR “mental disorder*” OR well-being OR Resilience OR behavior* OR prevention OR promotion)* ***AND*** *(child OR infant OR adolescent OR Puberty)* ***AND*** *(Iceland OR Ireland OR Israel OR Italy)*
7. *(Indicator* OR Monitoring OR Surveillance)* ***AND*** *(“mental health” OR “mental disorder*” OR well-being OR Resilience OR behavior* OR prevention OR promotion)* ***AND*** *(child OR infant OR adolescent OR Puberty)* ***AND*** *(Japan OR Korea OR Latvia OR Lithuania)*
8. *(Indicator* OR Monitoring OR Surveillance)* ***AND*** *(“mental health” OR “mental disorder*” OR well-being OR Resilience OR behavior* OR prevention OR promotion)* ***AND*** *(child OR infant OR adolescent OR Puberty)* ***AND*** *(Luxembourg OR Mexico OR Netherland)*
9. *(Indicator* OR Monitoring OR Surveillance)* ***AND*** *(“mental health” OR “mental disorder*” OR well-being OR Resilience OR behavior* OR prevention OR promotion)* ***AND*** *(child OR infant OR adolescent OR Puberty)* ***AND*** *(“New Zealand” OR Norway OR Poland)*
10. *(Indicator* OR Monitoring OR Surveillance)* ***AND*** *(“mental health” OR “mental disorder*” OR well-being OR Resilience OR behavior* OR prevention OR promotion)* ***AND*** *(child OR infant OR adolescent OR Puberty)* ***AND*** *(Portugal OR Slovakia OR Slovenia)*
11. *(Indicator* OR Monitoring OR Surveillance)* ***AND*** *(“mental health” OR “mental disorder*” OR well-being OR Resilience OR behavior* OR prevention OR promotion)* ***AND*** *(child OR infant OR adolescent OR Puberty)* ***AND*** *(Spain OR Sweden OR Switzerland OR Turkey)*
12. *(Indicator* OR Monitoring OR Surveillance)* ***AND*** *(“mental health” OR “mental disorder*” OR well-being OR Resilience OR behavior* OR prevention OR promotion)* ***AND*** *(child OR infant OR adolescent OR Puberty)* ***AND*** *(“United Kingdom”)*
13. *(Indicator* OR Monitoring OR Surveillance)* ***AND*** *(“mental health” OR “mental disorder*” OR well-being OR Resilience OR behavior* OR prevention OR promotion)* ***AND*** *(child OR infant OR adolescent OR Puberty)* ***AND*** *(“United States” OR OECD)*
